# Purification of rabbit serum histidine-proline-rich glycoprotein via preparative gel electrophoresis and characterization of its glycosylation patterns

**DOI:** 10.1371/journal.pone.0184968

**Published:** 2017-09-21

**Authors:** Anna Katharina Weyrauch, Mario Jakob, Angelika Schierhorn, Ralf Bernd Klösgen, Dariush Hinderberger

**Affiliations:** 1 Institute of Chemistry, Division of Physical Chemistry, Martin Luther University Halle-Wittenberg, Halle (Saale), Saxony-Anhalt, Germany; 2 Institute of Biology, Division of Plant Physiology, Martin Luther University Halle-Wittenberg, Halle (Saale), Saxony-Anhalt, Germany; 3 Institute of Biochemistry and Biotechnology, Service Unit for Mass Spectrometry, Martin Luther University Halle-Wittenberg, Halle (Saale), Saxony-Anhalt, Germany; Swiss Institute of Bioinformatics, SWITZERLAND

## Abstract

Histidine-Proline-rich Glycoprotein (HPRG) is a plasma protein of vertebrates and several marine bivalves. Due to its multidomain structure consisting of several regions HPRG can interact with a variety of ligands, however the exact physiological role has not been discovered yet. Past purification approaches out of plasma or serum often led to co-purification of other proteins so that for a profound understanding of the function it is important to obtain a protein of high purity. Recent purification strategies were based upon metale chelate affinity chromatography followed by anion exchange chromatography or size exclusion chromatography, respectively. A large amount of serum albumin, the major plasma protein, also elutes from metale chelate affinity chromatography columns. Separation of rabbit HPRG from rabbit serum albumin could not be achieved via the above named methods by us. We present a method of purification of rabbit serum HPRG by means of metal affinity chromatography and preparative gel electrophoresis, which makes it possible to obtain HPRG practically devoid of impurities as assessed by mass spectrometry analysis. Moreover, we characterize the amount of glycosylation of HPRG and–to the best of our knowledge for the first time–the glycosylation pattern of rabbit HPRG.

## Introduction

Histidine-Proline-rich Glycoprotein (HPRG), or alternatively Histidine-rich Glycoprotein (HRG), is a protein of vertebrate plasma [1, 2] that was recently also found in the plasma of several marine bivalves [3, 4]. First isolated and characterized in 1972 by Heimburger et al. [5, 6] the concentration in human plasma was found to be 100–150 mg/l, whereas in rabbit plasma a concentration of up to 900 mg/l was demonstrated [7]. The molecular weight of the fully glycosylated protein is about 70 kDa for mammalian species [2].

HPRG, alongside fetuins and kininogen, is a type 3 cystatin and therefore a member of the cystatin superfamily. However, only kininogen was so far shown to possess cysteine protease inhibitor activity. Like most of the other members of the type 3 subgroup HPRG is produced in the liver [8–11].

**Fig 1:**
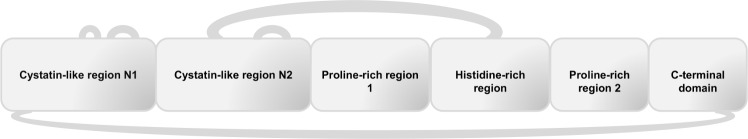
Schematic structure of HPRG. Schematic structure of the multiple domain structure of HPRG. Rabbit HPRG is supposed to possess five disulfide bridges according to [13].

As summarized in Fig 1, the protein consists of multiple domains and is stabilized by presumably five disulfide bonds. Besides the two Cystatin-like N-terminal regions (N1 and N2) HPRG possesses a central histidine-rich region (HRR), flanked by two proline-rich regions (PRR1 and PRR2) and a C-terminal domain. Both, the histidine-rich region and the two proline-rich regions are assumed to be intrinsically unstructured so that the full length protein is not crystallizable and hence not accessible to characterization by means of X-ray crystallography as the central method in structural biology. Nevertheless, as the two Cystatin-like domains are assumed to have more ordered secondary structures, Kassaar et al. [12] were able to crystallize the N2-domain.

The primary structure of the rabbit protein was long believed to be similar to that described for the human protein [7]. However, recently Ronca and Raggi [13] suggested an amino acid sequence that deviates from that published for the rabbit protein by Borza et al. in 1996 [7] [GenBank accession no. AAC48516.1] built upon a prediction of the transcribed RNA sequence [see GenBank accession no. XP_008264798.1]. Therefore, the actual location of the domains within the rabbit protein have to be reconsidered as proposed by Ronca and Raggi [13].

HPRG can interact with a variety of ligands and seems to be involved in the blood coagulation and fibrinolysis systems as well as in regulation of angiogenesis and interacts with components of the immune system [2, 14]. Yet, its exact physiological role still appears to be enigmatic. Due to the high content of histidines within the central histidine-rich region HPRG can interact with divalent metal ions and heme, although systematic studies on the metal binding capabilities are still remarkably scarce [15–18].

Other ligands that have been proven or are assumed to interact with HPRG include heparin [6, 19], heparanase [20], heparan sulfate [21], plasminogen [22], and thrombospondins-1 and -2 [23, 24]. The coarse and general protein structure, potential functions and ligands of the human protein have already been reviewed in the past years [1, 25].

Past purification approaches were mainly based upon phosphocellulose columns or nickel affinity chromatography. More recent purification strategies consisted of nickel affinity chromatography and size exclusion chromatography [12] and secondly cobalt affinity chromatography and anion exchange [26]. Patel et al. [26] stated that their purified protein showed no interaction with some ligands that were previously shown to interact with HPRG. This is an indication that in prior studies some ligands may not have interacted with HPRG but rather with co-purified proteins but final prove for this indication is certainly hard to achieve with one study alone.

Our finding was, when nickel/cobalt affinity chromatography was carried out, a large amount of serum albumin also eluted from the IMAC column (S1 Fig, S1 Table, S2 Table). Our efforts to separate serum albumin, as described by Patel et al. [26], with anion exchange could not successfully be adapted. The yields were low and respective bands on SDS-gel were weak. Moreover there were fragments visible running on the marker height of BSA (S2 Fig). This fragment was also obtained with size exclusion chromatography after metal chelate affinity chromatography (S3 Fig).

We hence developed a rather simple method based on the procedure described in Hauer et al. [27] which for the first time employs preparative gel electrophoresis to obtain rabbit HPRG in very high purity and practically devoid of albumin. By mass-spectrometric analysis, we can also–to the best of our knowledge for the first time–shed light on the glycosylation patterns of rabbit HPRG.

## Materials and methods

### Purification of rabbit serum HPRG

HPRG was purified from preservative free rabbit serum (Bio-Rad AbD Serotec, Kidlington, UK) at 4°C to prevent proteolytic degradation. Briefly, ten milliliters of serum aliquots were thawed and then incubated with 1 ml of PureCube Co-NTA (Biozym, Hessisch Oldendorf, Germany) for one to two hours. To prevent unspecific binding to the column material 20 mM imidazole was added. Further proteolytic degradation was inhibited by adding a protease inhibitor cocktail according to the manufacturer’s instructions (Inhibitor cocktail tissue, Carl Roth, Germany). The suspension was then transferred to a column, washed with wash buffers and HPRG was eluted with 500 mM imidazole. Buffers used were of the compositon according to [26] with minor changes regarding the protease inhibitor cocktail.

The elution fractions were analyzed via SDS-PAGE. Fractions containing HPRG were concentrated (Vivaspin 2, 10.000 MWCO, Sartorius, Göttingen, Germany) and the concentration was determined via BCA-Assay (Pierce^TM^ BCA Protein Assay Kit, ThermoFisher Scientific, Waltham, USA).

0.5–1 mg of the protein solution was diluted in Laemmli sample buffer [28] without reducing agents and separated on 10% SDS-gel (20 x 20 cm). The protein gel bands were visualized by non-fixing zinc-imidazole staining [29]. The band corresponding to HPRG was recovered and cut into small pieces. Electroelution was carried out at 70 V overnight using chambers filled with standard SDS running buffer. The protein was collected from the anodic side of the chambers and stored at 4°C. The precise specifications of the equipment for preparative gel electrophoresis and electroelution, the composition for buffers and gels as well as running conditions are summarized in S1 Appendix.

Finally the protein was applied to a reversed-phase HPLC (column EC 125/4 Nucleosil 500–5 C3-PPN, Macherey & Nagel; flow rate 1 ml/min; detection wave length: 220 nm) and eluted with an acetonitrile-water gradient. Fractions were collected after 19 min, HPRG eluted at about 23–24 min from the column. The fractions containing HPRG were lyophilized. The applied acetonitrile-water gradient is shown in Table 1.

**Table 1:** Acetonitrile-Water gradient applied to the reversed phase HPLC.

time / min	solvent A / %	solvent B / %
0–6	90	10
6–7	90–70	10–30
7–20	70–57	30–43
20–20.3	57–40	43–60
20.3–25	40	60
25–27	40–10	60–90
27–30	10	90
30–32	10–90	90–10
32–40	90	10

Acetonitrile-Water gradient applied to the reversed phase HPLC to purify HPRG from remaining buffer salts and SDS after preparative gel electrophoresis (solvent A: water + 0,05% TFA; solvent B: acetonitrile + 0,05% TFA). The sudden jump from 43% to 60% acetonitrile at 20.3 min focuses the protein to few elution fractions.

### SDS-PAGE

Protein samples of the cobalt affinity chromatography elution fractions were diluted in Laemmli sample buffer [28] without reducing agents and electrophoresed on 12% SDS-gels. Gels were stained with colloidal Coomassie Brilliant Blue G250 (Roti®-Blue, Carl Roth, Karlsruhe, Germany). MW size marker used was the Protein Marker III (6.5–200) (AppliChem, Darmstadt, Germany): Myosin (212 kDa), β-Galactosidase (118 kDa), BSA (66 kDa), Ovalbumin (45 kDa), Carbonic anhydrase (29 kDa), Trypsin inhibitor (soy bean) (20 kDa), Lysozyme (14 kDa), Aprotinin (bovine lung) (6.5 kDa).

### Determination of protein concentration

The final protein concentration after dissolving the lyophilized protein in buffer was determined via UV/Vis-spectroscopy. The molar extinction coefficient was calculated according to [30]:

$$\varepsilon_{280}\;[M^{-1}{cm}^{-1}]=5500*nW+1490*nY+62.5*nC$$

$$\varepsilon_{280}\;[M^{-1}{cm}^{-1}]=5500*3+1490*10+62.5*12=32150\;M^{-1}{cm}^{-1}$$

### Mass spectrometry

For mass spectrometry (MS) analysis 50 μg of the lyophilized protein were dissolved in 50 mM NH_4_HCO_3_ buffer and 0.1% ProteaseMAX^TM^ Surfactant (Promega) was added according to the manufacturer’s instructions. A small fraction of this preparation was used for determination of the molecular weight by MALDI MS.

To alkylate the cysteine residues the protein was reduced (0.5 M dithiothreitol (Sigma), 20 min, 56°C), incubated with 0.55 M iodoacetamide (Sigma) for 15 min in the dark, and then incubated overnight with trypsin (Promega) and Asp-N (Promega), respectively.

To further analyze the protein regarding the glycosylation sites the carbohydrates were removed in a parallel preparation by treatment with PNGase F (Promega) for 4h at 37°C. 0.1% ProteaseMAX^TM^ Surfactant was added according to the manufacturer’s instructions. Again a small fraction of this preparation was used for determination of the molecular weight by MALDI MS. After deglycosylation the protein solution was digested overnight with trypsin (Promega) and Asp-N (Promega) at room temperature, respectively.

For MALDI-TOF-MS analysis 1 μl of a solution of 2.5-dihydroxy-benzoic acid in methanol (7mg/100μl) was mixed with 1 μl protein solution and 1 μl of the mixture was deposited onto a stainless steel target.

The protein spectra were recorded on an Ultraflex-II TOF/TOF mass spectrometer (Bruker Daltonic, Bremen, Germany) equipped with MALDI source, nitrogen laser, LIFT cell for fragment ion postacceleration and gridless ion reflector. The software Flex Control 3.0, Flex Analysis 3.0 and Biotools 3.0 were used to operate the instrument and analyze the data. For external calibration a protein calibration mixture (Bruker Daltonics, Bremen, Germany) was used.

For ESI-QTOF-MS/MS measurements the peptide solution was injected into a nanoACQUITY UPLC system (Waters Co.) equipped with a binary solvent manager, sample manager and heating and trapping module. 2 μl were injected via “microliter pickup” mode and desalted on-line through a symmetry C18 180μm×20mm precolumn. The peptides were separated on a 100μm×100mm analytical RP column (1.7μm BEH 130 C18, Waters Co.) using a typical UPLC gradient from 3.0% to 33.0% over 15min. The mobile phases used were 0.1% formic acid in water and 0.1% formic acid in acetonitrile. The column is connected to an SYNAPT^®^ G2 HDMS- mass spectrometer (Waters Co.), which is a hybrid quadrupole tandem time-of-flight (Q-TOF) mass spectrometer, equipped with Tri-wave ion guides that trap and separate ions by ion mobility (Waters Co.). The data were acquired in LC/MS^E^ mode switching between low and elevated energy on alternate scans. Subsequent correlation of precursor and product ions can then be achieved using retention time alignment.

BiopharmaLynx (1.3.2, Waters Co.) was used to analyze the obtained MS and MS/MS data. Searches were conducted with GlobalSERVER™ using SwissProt database.

### Circular dichroism

CD measurements were performed on a Jasco J-810 CD spectrometer at 20°C (. The wavelength range was set to values where the dynode voltage did not exceed 600 V (190–250 nm). Measurements were carried out at a protein concentration of 0.69 mg/ml (10 mM phosphate, 50 mM NaCl, pH 7.4) in a 0.1 mm cuvette, 60 scans were averaged. CD data were analyzed with the Dichroweb Tool [31].

## Results and discussion

### Purification of rabbit serum HPRG

HPRG is a plasma glycoprotein that occurs in many vertebrates. Standard expression protocols for purifying the native protein from E. coli do not work because many bacteria, including E coli, are not able to glycosylate. Hence, in order to obtain the native protein, purifying it out of plasma or serum seems the better choice. As already pointed out in the introduction there were already a few purification strategies published which led to co-purification of other proteins. More recent purification strategies were based upon metal chelate affinity chromatography and ion exchange chromatography or size exclusion chromatography, respectively. However in our case we were not able to separate the rabbit serum albumin, which did also elute during metal chelate affinity chromatography.

**Fig 2:**
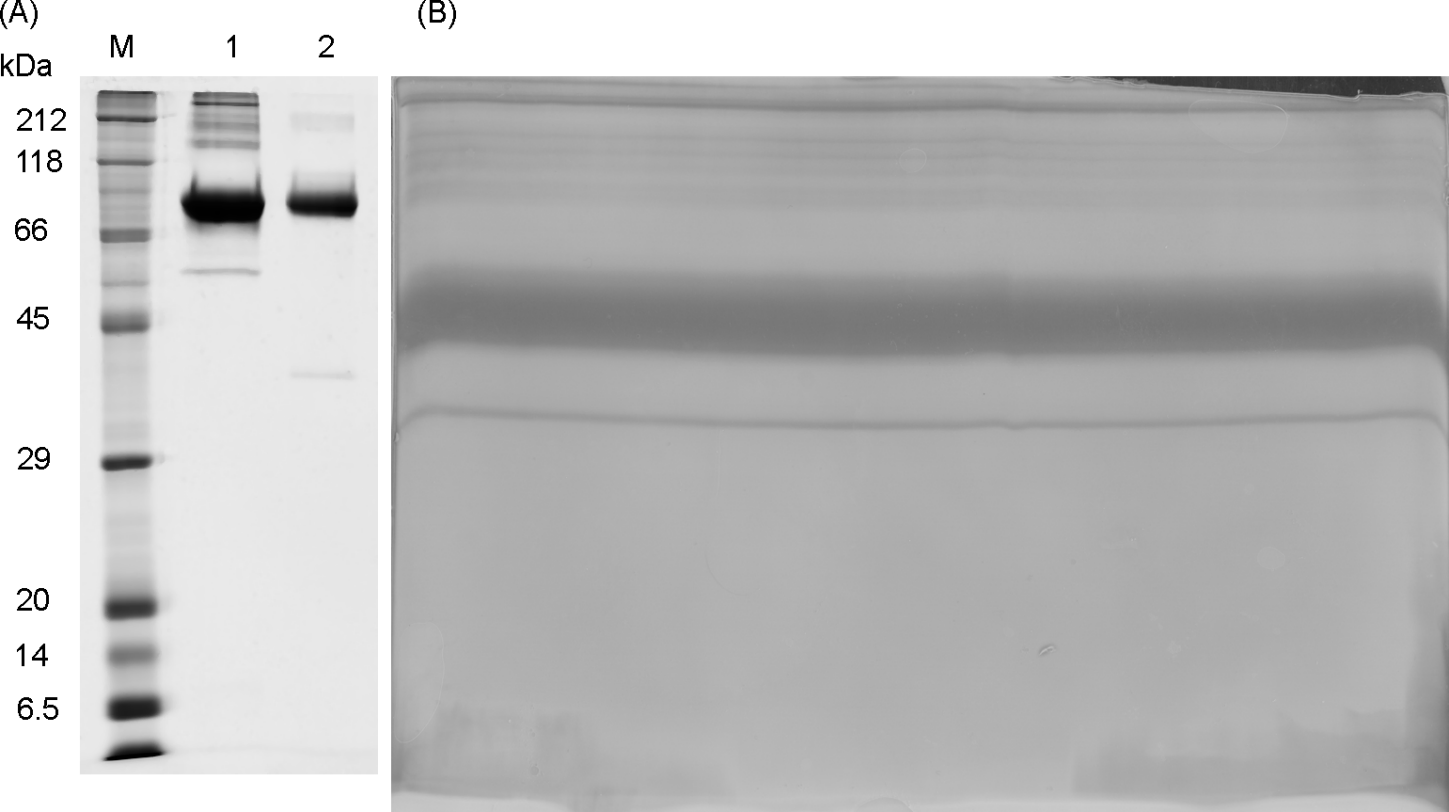
**A and B. SDS-PAGE and zinc-imidazole stained midi gel.** (A) SDS-PAGE of two elution fractions of the cobalt-affinity chromatography in lane 1 and 2. In lane M the marker is shown. HPRG migrates on SDS-Gel at approximately 90 kDa. Minor proteolytic fragments with lower molecular weight are present. In lane 2 the lower band may also correspond to serum albumin (B) Zinc-imidazole stained gel after preparative gel electrophoresis. The thick band corresponds to the native HPRG and is cut out.

We here introduce an alternative purification approach with the first step being identical to the cobalt-affinity chromatography approach published by Patel et al. [26]. Due to the high content of histidines within the histidine-rich region, where 33 histidines of the overall 60 histidines are located [13], this region can act as a kind of “natural his-tag” and can thus be utilized for metal chelate affinity chromatography. As we noticed that the respective HPRG gel band is well-separated from other co-purified proteins and fragments, we utilized preparative gel electrophoresis of the obtained elution fractions. The concentration of the protein in rabbit plasma is about sixfold higher compared to that in human plasma, so we conducted this first trial with the rabbit protein, notwithstanding application of the procedure to the human analogue. Since HPRG is known to be sensitive to protease degradation, especially to plasmin cleavage [32], we performed the cobalt affinity chromatography in a cold room at 4°C. To further minimize proteolytic degradation a protease inhibitor cocktail was added to the incubation mixture. A smaller portion was also added to the wash buffers. After elution of the protein from the column with 500 mM imidazole the fractions were analyzed via SDS-PAGE (Fig 2A).). The fractions containing the protein were concentrated by a MWCO of 10.000 Da at 4°C.

The major impurity that needs to be removed is serum albumin. Serum albumin migrates differently on SDS-gels depending on electrophoresis conditions, e.g. if it is reduced (apparent size of 67 kDa) or not reduced (apparent size about 55 kDa) [33, 34]. This is the reason why under the conditions used in our preparative gel electrophoresis approach (SDS-PAGE under non-reducing conditions) it is possible to separate serum albumin from HPRG. Rabbit HPRG migrates at about 90 kDa on SDS-PAGE under reducing conditions [7]. In this work the 12% SDS gels following cobalt affinity chromatography were carried out at non-reducing conditions in order to have the same conditions at this step as in the later preparative gel electrophoresis for a better comparison. Interestingly the rabbit HPRG does not seem to be strongly affected if the SDS-PAGE is under reducing or non-reducing conditions. This may be due to the large region with intrinsic disorder that the protein is already stretched.

The gel size for preparative gel electrophoresis was chosen to Midisize (20 x 20 cm) in order to be able to load a higher amount of the protein solution (about 0,5–1 mg protein). To separate HPRG from serum albumin as already stated above and to guarantee that disulfide bridges are not reduced, reducing agents were not used. For negative staining, zinc-imidazole staining was chosen (Fig 2B). After cutting out the gel band belonging to HPRG it was sliced in small pieces and electroeluted with standard SDS running buffer out of the slices.

We also tested native electrophoresis without SDS and without reducing agents (data not shown). In these, however, the protein did not elute from the gel slices during electroelution demonstrating that at least SDS is necessary to effectively elute the protein to the anodic side of the electroelution chamber.

To remove SDS and buffer salts from electroelution a reversed-phase HPLC was carried out at room temperature. HPRG fractions were pooled and lyophilized.

The overall yield of rabbit HPRG during cobalt affinity chromatography is about 30% if the concentration of HPRG in plasma is correctly determined to 900 mg/l as stated in Borza et al. [7]. For the human HPRG isolated from plasma via Co-IMAC by Patel et al. [26] and whose buffer compositions were used in this work the recovery was 55.95% within this first step. In order to obtain a good separation during preparative gel electrophoresis a sample amount of 0.5–1 mg after determination via BCA was loaded onto the gel. No significant loss of HPRG should be in this purification step so that the recovery for gel electrophoresis and subsequent electroelution is > 90%. However it has to be kept in mind that the protein concentration determined via BCA include impurities that are separated via gel electrophoresis and electroelution. The recovery after RP-HPLC is higher than 80%.

### Mass spectrometry

The high purity of the HPRG preparation could be confirmed by mass spectrometry. Only approximately 1% contamination with rabbit serum albumin was detected in the tryptic digest of the lyophilized protein after nanoLC-ESI-MS/MS analysis (S3 Table). Disulfide bonds were reduced and the cysteine residues alkylated with iodoacetamide before every digestion.

The results of the HPRG tryptic digest (data not shown) were matched against both published sequences of rabbit HPRG AAC48516.1 (published by Borza et al. [7]) (51.7% sequence coverage) and the more recently published sequence XP_008264798.1 [13] (62.2% sequence coverage). Our data are in better agreement with the more recently published sequence, therefore we used XP_008264798.1 for MS-data interpretation.

As HPRG is known to be a glycoprotein [2], the glycans have to be removed from the protein to allow verification of the amino acid sequence. HPRG was incubated with PNGase F, a N-glycosidase that catalyzes the cleavage of N-linked oligosaccharides, before digestion with trypsin or Asp-N. Digestion with Asp-N as a second protease, which cleaves peptide bonds on the N-terminal site of aspartic acid, increased the sequence coverage. For a complete coverage analysis, all peptides were combined. The N-terminal peptides AS 3–24 (tryptic digest) and AS 1–22 (Asp-N digest) were missing. De novo sequencing of an unmatched peptide with *m/z* 834.40 led to the sequence LTPTDC_mod_K, matching amino acids 19–25 of the HPRG sequence (S4 Fig).

**Fig 3:**
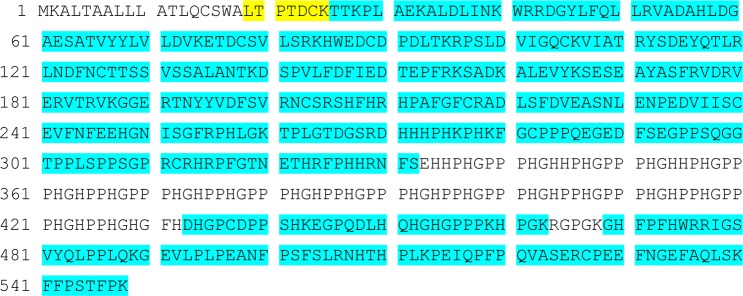
HPRG sequence coverage. Total sequence coverage (79.6%) of HPRG (sequence XP_008264798.1) combining results of trypsin and Asp-N digests of HPRG after PNGase F treatment. Turquoise: matched amino acids. Yellow: de novo sequenced peptide.

HPRG is a plasma protein and thus needs to be secreted. The N-terminal 18 amino acids of the rabbit HPRG sequence are therefore likely to be part of the N-terminal signal peptide needed for secretion that is removed during transport. Another missing part of the HPRG amino acid sequence is the central histidine-rich region, because there are no restriction sites available for proteases. Taking the results of the trypsin and Asp-N digestion almost 80% sequence coverage could be achieved (Fig 3).

**Fig 4:**
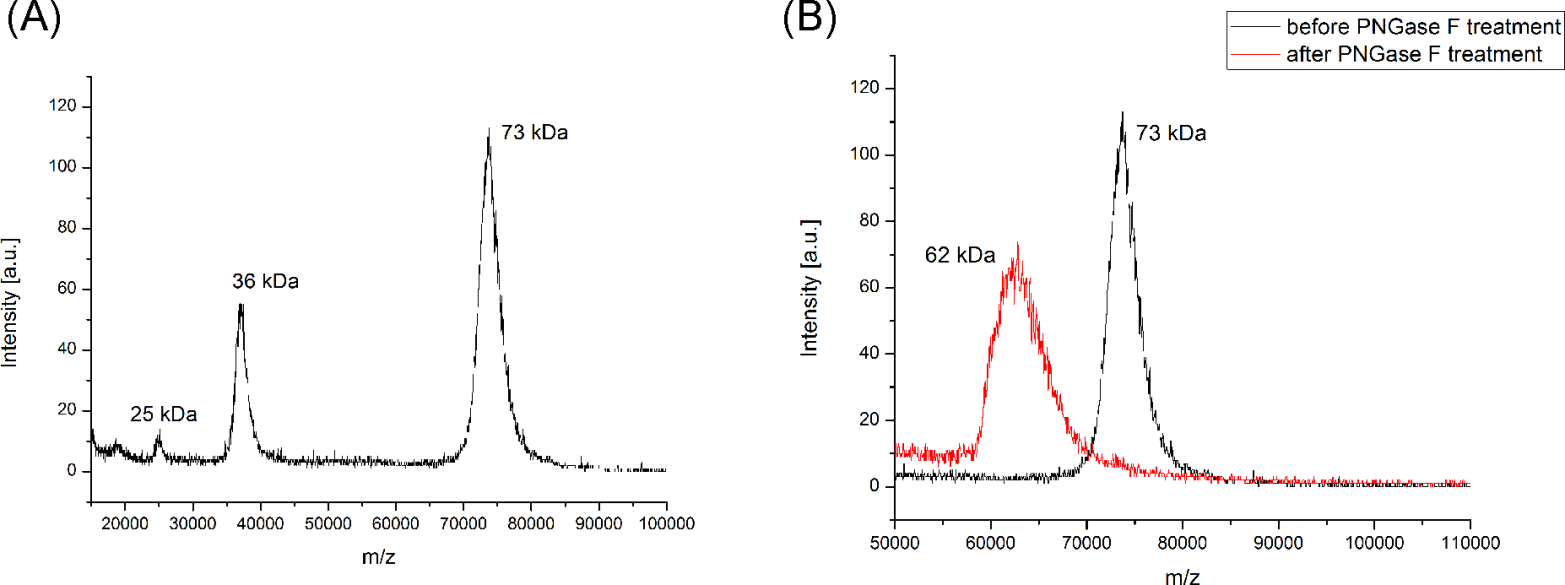
**A and B. MALDI-TOF-MS spectra.** (A) MALDI-TOF-MS spectrum of the rabbit HPRG before PNGase F treatment shows that the isolated protein has no major impurities. The peak at 73 kDa corresponds to the native protein. The peaks at 35 kD and 25 kDa are the [M+2H]^2+^ and [M+3H]^3+^ ions. (B) MALDI-TOF-MS spectra of the HPRG before treatment with PNGase F (black), and after PNGase F treatment (red).

The lyophilized protein recovered after preparative gel electrophoresis has a molecular weight of 73 kDa, as determined by MALDI-TOF-MS, which is consistent with the molecular weight of about 70 kDa for the glycosylated protein described by Wakabayashi [2]. After PNGase F digestion, a molecular weight of 62 kDa was discovered, thus the glycosylation amounts to about 11 kDa (Fig 4A and 4B).

There are six theoretical N-linked glycosylation sites (N-X-S/T motif, where X can be any amino acid except proline) in the HPRG sequence, N-125, N-202, N-250, N-320, N-330, and N-507. For five of the six potential glycosylation sites glycopeptides could be found analyzing the trypsin and Asp-N digests without PNGase F treatment using nanoLC-ESI-MS/MS. The only missing glycosylation site N-330 belongs to the tryptic peptide AS 330–443 (MW 11883.5061Da) and Asp-N peptide AS 290–432 (MW 15124.1251Da) with the histidine rich sequence, which were not detected in any of the digests. A summary of all identified glycopeptides is shown in Table 2.

**Table 2:** Summary of N-glycosylation mapping of rabbit HPRG by nanoLC-ESI-MS/MS.

Glyco site	Digest	Glycan composition	Start AS	End AS	Modification	RT (min)	Intensity (Counts)	Sequence	Observed GP Mass (MW)	Theoretical GP Mass (MW)
**N 125**	Trypsin	2*(NeuAc), 4*(HexNac),5*(Hex)	121	139	Carbamidomethyl C(1)	18.9	436622	LNDF**N**CTTSSVSSALANTK	4233.7261	4233.7146
	AspN	2*(NeuAc), 4*(HexNac),5*(Hex)	123	139	Carbamidomethyl C(1)	18.2	643631	DF**N**CTTSSVSSALANTK	4006.5920	4006.5876
**N 202**	AspN	2*(NeuAc), 4*(HexNac),5*(Hex)	197	219	Carbamidomethyl C(2)	15.4	42206	DFSVR**N**CSRSHFHRHPAFGFCRA	5052.0918	5052.0818
**N 250**	AspN	3*(NeuAc), 5*(HexNac),6*(Hex)	235	265	Carbamidomethyl C(1)	22.1	19285	DVIISCEVFNFEEHG**N**ISGFRPHLGKTPLGT	6330.7212	6330.7088
	AspN	2*(NeuAc), 4*(HexNac),5*(Hex)	235	269	Carbamidomethyl C(1)	21.0	715358	DVIISCEVFNFEEHG**N**ISGFRPHLGKTPLGTDGSR	6089.6777	6089.6628
	AspN	2*(NeuAc), 4*(HexNac),5*(Hex)	235	265	Carbamidomethyl C(1)	21.8	284361	DVIISCEVFNFEEHG**N**ISGFRPHLGKTPLGT	5674.4956	5674.4812
**N 320**	Trypsin	3*(NeuAc), 5*(HexNac),6*(Hex)	314	324		12.6	148846	HRPFGT**N**ETHR	4211.6631	4211.6541
	Trypsin	2*(NeuAc), 4*(HexNac),5*(Hex)	314	324		12.3	897180	HRPFGT**N**ETHR	3555.4302	3555.4265
**N 507**	Trypsin	3*(NeuAc), 5*(HexNac),6*(Hex)	507	526		17.7	346476	**N**HTHPLKPEIQPFPQVASER	5185.2119	5185.2026
	Trypsin	2*(NeuAc), 4*(HexNac),5*(Hex)	507	526		17.3	2017335	**N**HTHPLKPEIQPFPQVASER	4528.9741	4528.9750

**Fig 5:**
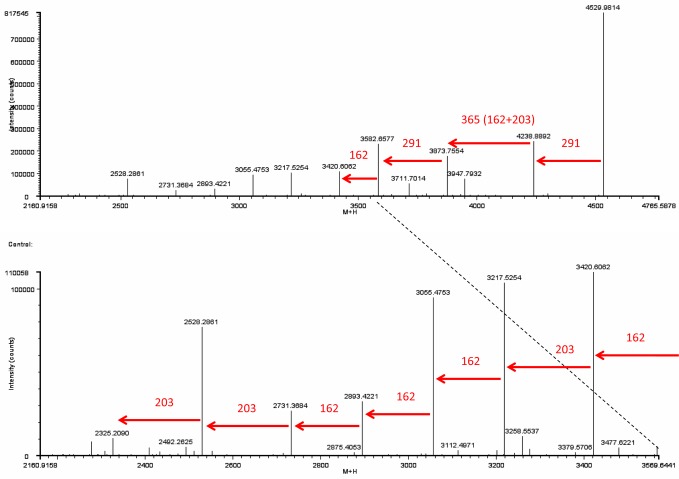
MS/MS-spectrum of glycopeptide AS 507–526. Detail of the MS/MS-spectrum (deconvoluted to singly charged ions) of glycopeptide AS 507–526 NHTHPLKPEIQPFPQVASER (trypsin digest) (glycosylation site N-507). The precursor ion *m/z* 4529.9814 was successively fragmented due to the loss of 2 NeuAc, 4 HexNAc and 5 Hex (glycan mass 2204.7724) Monosaccharid masses: NeuAc 291, HexNAc 203, Hex 162.

An example for the glycan fragmentation of the HPRG glycopeptides is given in Fig 5.

**Fig 6:**
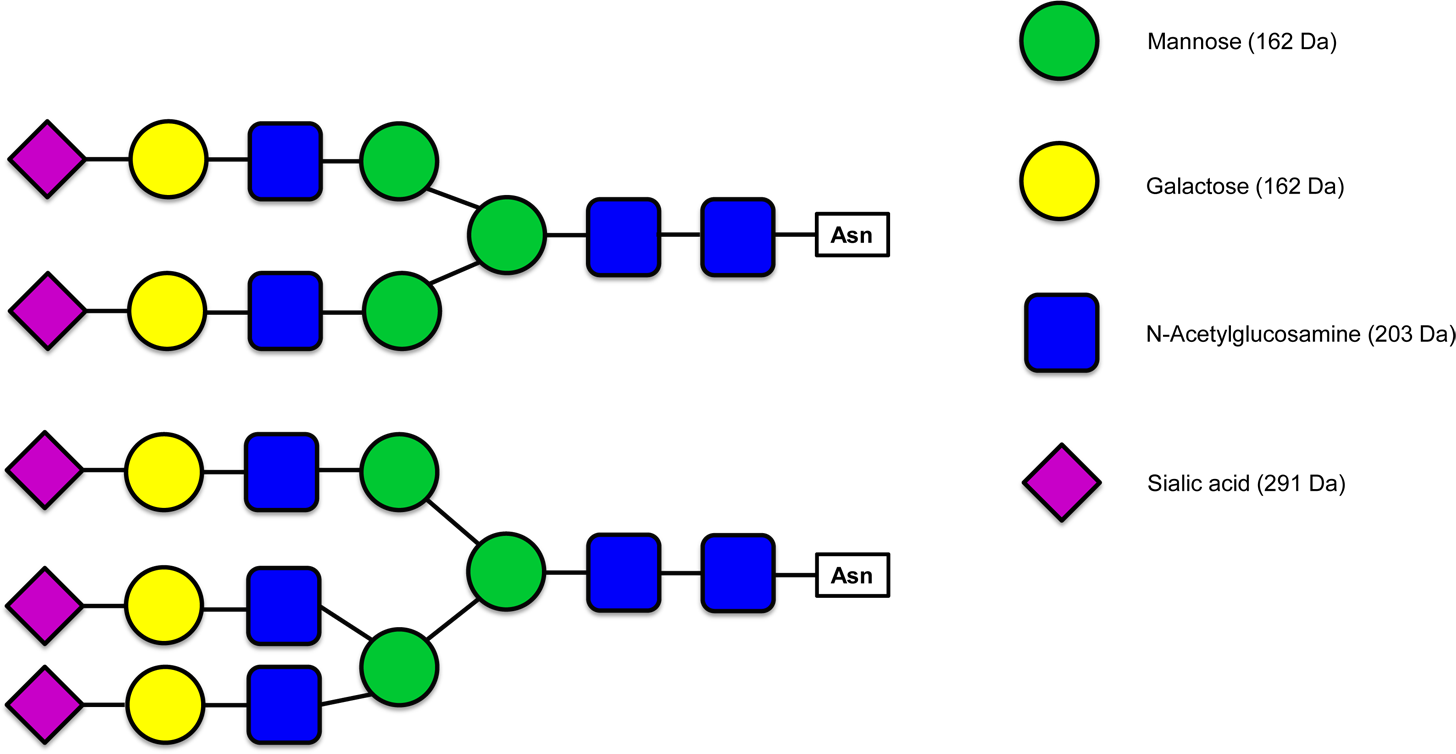
Possible structures of the complex N-glycans of rabbit HPRG. Glycan symbols are according to Essentials of Glycobiology/Consortium of Functional Glycomics nomenclature.

Fig 5 shows a detail of the MS/MS-spectrum of glycopeptide AS 507–526 NHTHPLKPEIQPFPQVASER (glycosylation site N-507). The peptide was found with a charge state of +4 (*m/z* 1133.2515) and a retention time of 17.3 min. The MS/MS-spectrum was deconvoluted to singly charged ions. The precursor ion 4529.9814 ([M+H]^+^) was successively fragmented due to the loss of NeuAc (291) followed by HexNAc (203) plus Hex (162) and again NeuAc (291). The glycan composition with 2 NeuAc, 4 HexNAc and 5 Hex (mass 2204.7724) indicates a complex type of N-linked glycan with biantennary structure (Fig 6). The difference between the measured mass of the glycopeptide and the mass of the glycan was 2324.1976 Da which is in agreement with the MW of peptide AS 507–526, seen as a small singly charged fragment ion (*m/z* 2325.2346) in the tandem mass spectra. This peptide could also be found with an added triantennary glycan (mass 2861.0) (Fig 6) at 17.7 min retention time with a MW of 5185.2119 Da. The glycosylation sites N-250 and N-320 also possess these two complex glycan structures. The glycopeptides with triantennary N-glycan forms have increased retention times (0.3–0.4 min difference). The third negatively-charged NeuAc leads to tighter binding to the reversed phase material [35]. MS/MS spectra of the other glycopeptides can be found in S5–S9 Figs. Collision induced fragmentation of glycopeptides mainly results in fragmentation of the sugar moieties but not the peptide. The deglycosylated peptides were identified in the trypsin and Asp-N digests after PNGase F treatment (S4 and S5 Tables). It is worth to note that after deglycosylation with PNGase F the asparagine residue to which the carbohydrate was attached is changed to an aspartic acid residue (N/D-change) and can thus be identified as glycosylation site. For Asp-N a new cleavage site is thereby created. So in the Asp-N digest of the deglycosylated HPRG the new peptides AS 202–219 (MW 2243.9966 Da), AS 250–265 (MW 1694.9104 Da) and AS 250–269 (MW 2110.0918 Da) occurred. For the N-glycosylation sites N-125, N-202, N-250, N-320 and N-507 no unglycosylated peptides were found in the trypsin and Asp-N digest without PNGase F treatment.

### Circular dichroism

Circular dichroism spectroscopy was carried out to obtain information about the protein’s secondary structure after dissolving the lyophilized protein in 10 mM phosphate, 50 mM NaCl, pH 7.4. Upon usage of a 0.1 mm cuvette and a sample volume of 50 μl the CD spectrum was recorded from 250 nm– 190 nm.

**Fig 7:**
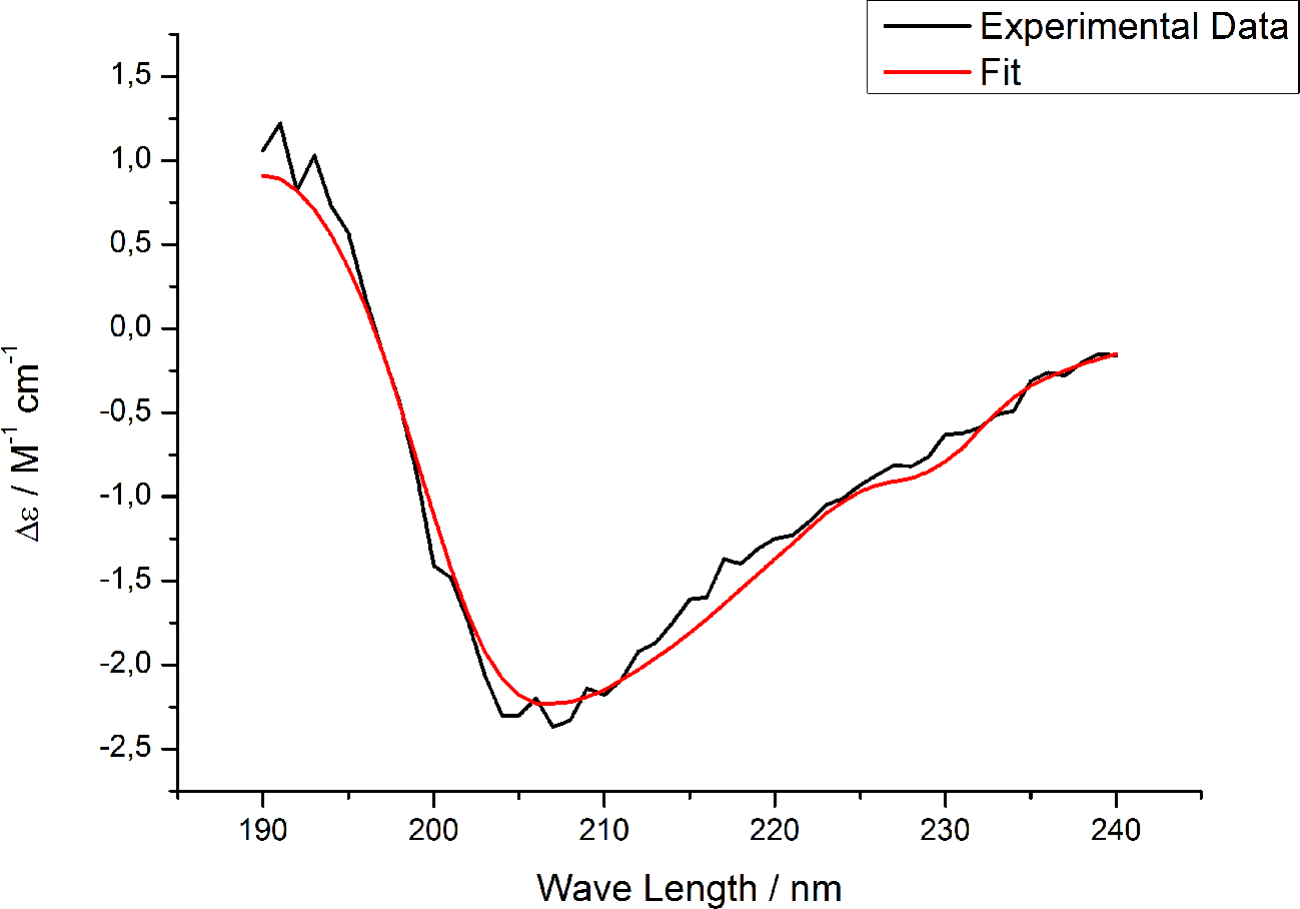
CD spectrum and CONTIN-Fot against reference dataset 7 [38, 39] of the rabbit HPRG (0.69 mg/ml in 10 mM phosphate, 50 mM NaCl, pH 7.4) in a 0.1 mm cuette cuvette. Δε was calculated with our finding that the protein consists of 530 amino acid residues and has a molecular weight of 73 kDa so that the mean residue weight is 138 Da.

Fig 7 shows the obtained spectrum for a concentration of 0.69 mg/ml in a 0.1 mm cuvette. The best fit was obtained when the spectrum was analyzed with the CONTIN algorithm [36, 37] against the reference dataset 7 [38, 39]. The closest matching solution yielded a content of 15.4% α-helix, 24.1% β-strand, 18.4% turns, and 42.1% unordered structure. This is already a good approximation of what is known about the protein having large amounts of disordered central regions, but ordered structure especially in the N-terminal cystatin-like domain [12]. Since reducing agents were not used during preparative gel electrophoresis and thus the disulfide bridges were kept intact the overall structure of the protein should have been preserved. But it has also to be kept in mind that we had to use SDS during preparative gel electrophoresis due to the fact that the anionic charge was needed to effectively elute the protein from the gel slices during electroelution so that there may be a few aggregates in the solution but may also be aggregates arising from lyophilization. Nevertheless our obtained CD data correspond well to the already published CD data [26]. However we are not able to obtain results whether there are 3/10-helices or Poly-Pro-II-helical structures due to the fact that we could only obtain data down to 190 nm. The DichroWeb reference sets [38, 39] that could analyse these secondary structures require CD data down to 176 nm. Due to poor signal to noise ratios when the dynode voltage during the measurements is too high it was not possible to determine reliable data at those low wavelengths.

## Conclusions

A major issue of purification of rabbit serum HPRG via metal chelate affinity chromatography is the co-purification of rabbit serum albumin. We present a purification approach to obtain rabbit serum HPRG by means of preparative gel electrophoresis that in a rather easy to perform procedure yields protein with only a minor impurity of about 1% of rabbit serum albumin. It is possible to isolate a pure protein, whose CD data matches well with the already published one however due to usage of SDS the solution may contain some aggregates. For studies regarding mass spectrometric analysis this method should work well. We are also able to show that our data correspond well with the recently published amino acid sequence of rabbit HPRG. By mass spectrometry analysis we characterize the amount of glycosylation and by collision-induced tandem MS we could show the glycosylation pattern of the protein which to the best of our knowledge was not known before. For studies regarding ligand binding it has to be kept in mind that the overall tertiary structure probably has not been compromised in our approach, because disulfide bridges were not reduced. For binding studies or studies on the structure of the protatin caution is recommended, nonetheless.

## Supporting information

S1 Fig Coomassie-stained SDS-gel of nickel affinity chromatography. Three consecutive elution fractions of nickel affinity chromatography (His Trap HP, GE Healthcare) are shown in lanes 2, 3, and 4. HPRG was eluted with 400 mM imidazole, as described in [12]. The band about 55 kDa could be shown to be serum albumin (S2 Table).
(TIF)

S2 Fig Coomassie-stained reducing SDS-gel of anion exchange chromatography. SDS-PAGE of elution fractions from anion exchange chromatography (Hi Trap Q FF, GE Healthcare). Yields are very low in lane 4, 5, and 6. A fragment running at the marker height (lane 1) of BSA is visible. For the anion exchange the cobalt affinity chromatography elution sample was diluted in 20 mM Tris, pH 8.5. The column was washed with the same buffer and HPRG eluted with 20 mM Tris,500 mM NaCl, pH 8.5.
(TIF)

S3 Fig Coomassie-stained reducing SDS-gel of gel filtration. SDS-PAGE of gel filtration (Superdex 200 10/300 GL, GE Healthcare). Again there is a fragment visible at the marker height (lane 1) of BSA. The size exclusion chromatography column was equilibrated with 25 mM phosphate, 154 mM NaCl, pH 7.4. The applied sample was the elution fraction of the cobalt affinity chromatography.
(TIF)

S4 Fig MS/MS-spectrum of peptide AS 19–25 (*m/z* 834.40) with the sequence LTPTDC_mod_K (mod = carbamidomethyl). (TIF)

S5 Fig Detail of the MS/MS-spectrum (deconvoluted to singly charged ions) of glycopeptide AS 123–139 (Asp N digest) (glycosylation site N-125). The precursor ion 4007.6001([M+H]^+^) was successively fragmented due to the loss of 2 NeuAc, 4 HexNAc and 5 Hex (glycan mass 2204.7724) Monosaccharid masses: NeuAc 291, HexNAc 203, Hex 162.
(TIF)

S6 Fig Detail of the MS/MS-spectrum (deconvoluted to singly charged ions) of glycopeptide AS 197–219 (Asp N digest) (glycosylation site N-202). The precursor ion 5053.1011([M+H]^+^) was successively fragmented due to the loss of 2 NeuAc, 4 HexNAc and 5 Hex (glycan mass 2204.7724) Monosaccharid masses: NeuAc 291, HexNAc 203, Hex 162.
(TIF)

S7 Fig Detail of the MS/MS-spectrum (deconvoluted to singly charged ions) of glycopeptide AS 235–265 (Asp N digest) (glycosylation site N-250). The precursor ion 5675.5103 ([M+H]^+^) was successively fragmented due to the loss of 2 NeuAc, 4 HexNAc and 5 Hex (glycan mass 2204.7724) Monosaccharid masses: NeuAc 291, HexNAc 203, Hex 162.
(TIF)

S8 Fig Detail of the MS/MS-spectrum (deconvoluted to singly charged ions) of glycopeptide AS 314–324 (trypsin digest) (glycosylation site N-320). The precursor ion 3556.4355 ([M+H]^+^) was successively fragmented due to the loss of 2 NeuAc, 4 HexNAc and 5 Hex (glycan mass 2204.7724) Monosaccharid masses: NeuAc 291, HexNAc 203, Hex 162.
(TIF)

S9 Fig Detail of the MS/MS-spectrum (deconvoluted to singly charged ions) of glycopeptide AS 507–526 (trypsin digest) (glycosylation site N-507). The precursor ion 5186.2197([M+H]^+^) was successively fragmented due to the loss of 3 NeuAc, 5 HexNAc and 6 Hex (glycan mass 2861.0000) Monosaccharid masses: NeuAc 291, HexNAc 203, Hex 162.
(TIF)

S1 Table ESI-MS-data results from nickel affinity chromatography via His Trap HP, GE Healthcare. (PDF)

S2 Table In-Gel-Digest result of 55 kDa band (lane 2) of S1 Fig. (PDF)

S3 Table ESI-MS-data results of the lyophilized HPRG sample after preparative gel electrophoresis. (PDF)

S4 Table nanoLC-ESI-MS/MS results from the tryptic digest of HPRG after PNGase F treatment. (PDF)

S5 Table nanoLC-ESI-MS/MS results from the Asp-N digest of HPRG after PNGase F treatment. (PDF)

S1 Appendix Manual for preparative gel electrophoresis and electroelution. (PDF)
