# Supplementary figures and images for: Purification of rabbit serum histidine-proline-rich glycoprotein via preparative gel electrophoresis and characterization of its glycosylation patterns
Source: PLoS One. 2017 Sep 21;12(9):e0184968. doi: 10.1371/journal.pone.0184968 (PMC5608300; doi:10.1371/journal.pone.0184968)

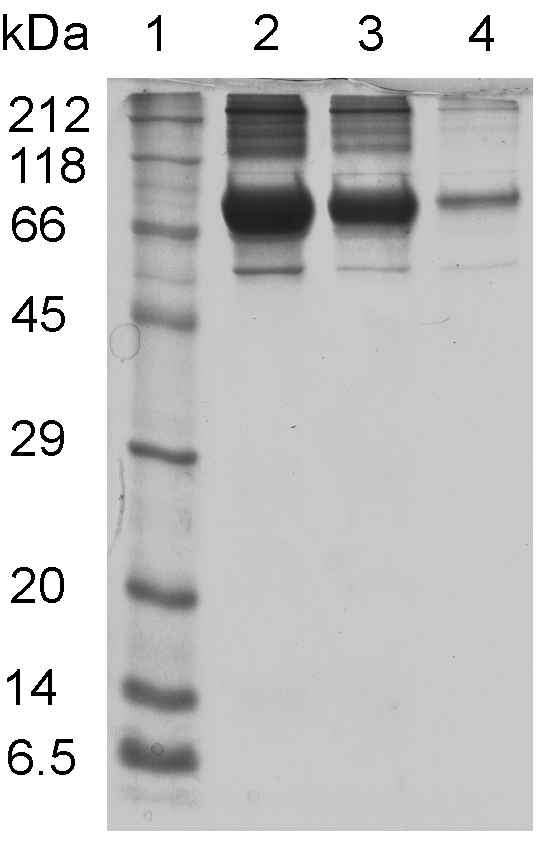

Supplement: S1 Fig — Three consecutive elution fractions of nickel affinity chromatography (His Trap HP, GE Healthcare) are shown in lanes 2, 3, and 4. HPRG was eluted with 400 mM imidazole, as described in [12]. The band about 55 kDa could be shown to be serum albumin (S2 Table). (TIF) [file pone.0184968.s001.tif]

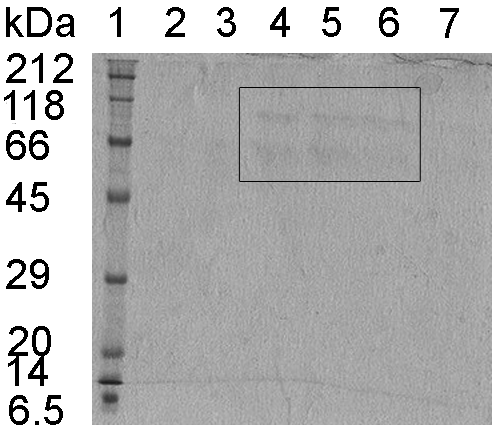

Supplement: S2 Fig — SDS-PAGE of elution fractions from anion exchange chromatography (Hi Trap Q FF, GE Healthcare). Yields are very low in lane 4, 5, and 6. A fragment running at the marker height (lane 1) of BSA is visible. For the anion exchange the cobalt affinity chromatography elution sample was diluted in 20 mM Tris, pH 8.5. The column was washed with the same buffer and HPRG eluted with 20 mM Tris,500 mM NaCl, pH 8.5. (TIF) [file pone.0184968.s002.tif]

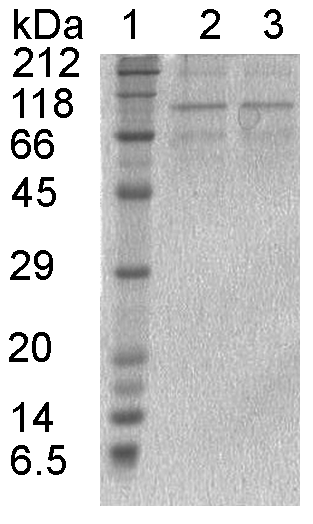

Supplement: S3 Fig — SDS-PAGE of gel filtration (Superdex 200 10/300 GL, GE Healthcare). Again there is a fragment visible at the marker height (lane 1) of BSA. The size exclusion chromatography column was equilibrated with 25 mM phosphate, 154 mM NaCl, pH 7.4. The applied sample was the elution fraction of the cobalt affinity chromatography. (TIF) [file pone.0184968.s003.tif]

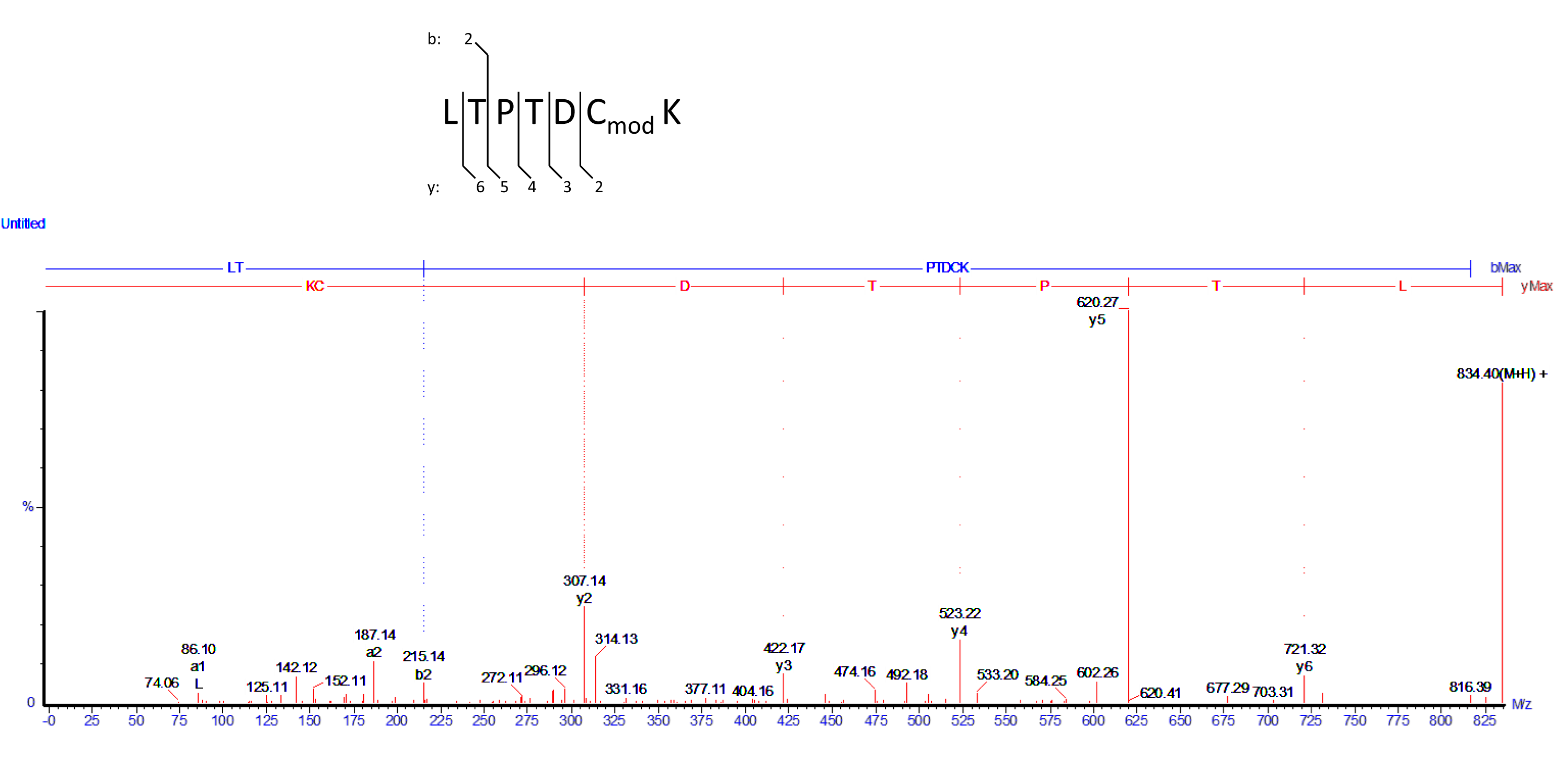

Supplement: S4 Fig — (TIF) [file pone.0184968.s004.tif]

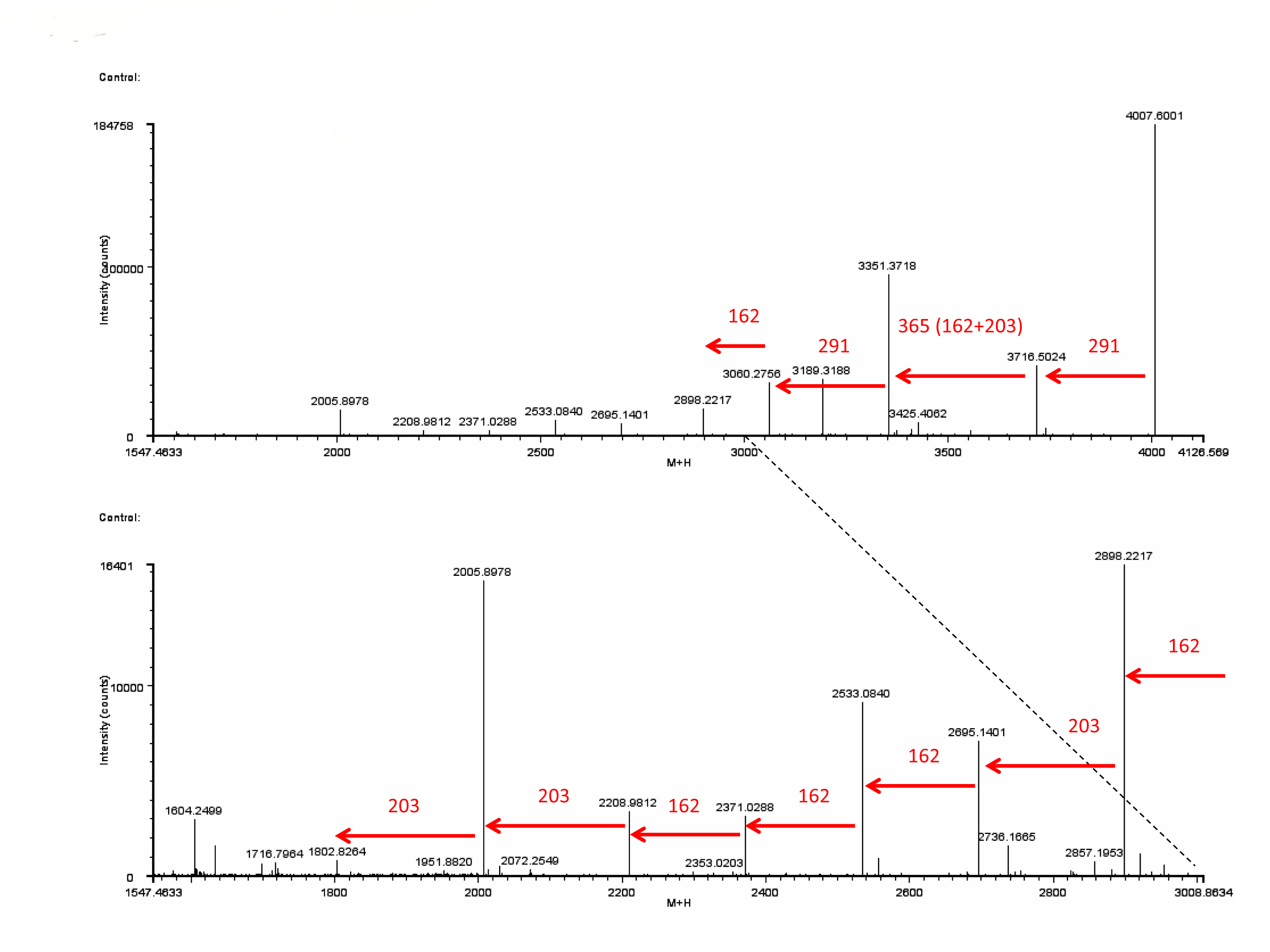

Supplement: S5 Fig — The precursor ion 4007.6001([M+H]+) was successively fragmented due to the loss of 2 NeuAc, 4 HexNAc and 5 Hex (glycan mass 2204.7724) Monosaccharid masses: NeuAc 291, HexNAc 203, Hex 162. (TIF) [file pone.0184968.s005.tif]

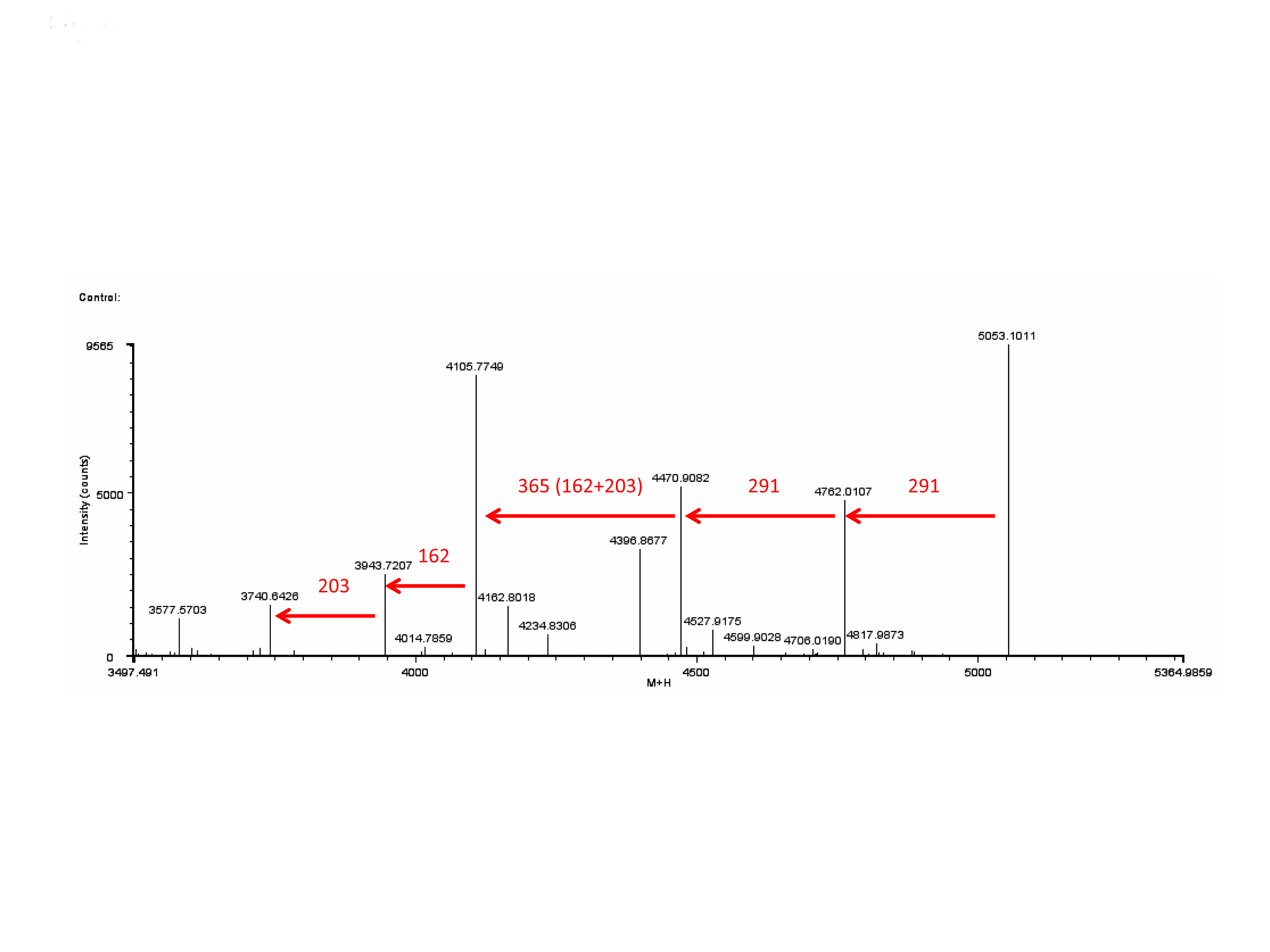

Supplement: S6 Fig — The precursor ion 5053.1011([M+H]+) was successively fragmented due to the loss of 2 NeuAc, 4 HexNAc and 5 Hex (glycan mass 2204.7724) Monosaccharid masses: NeuAc 291, HexNAc 203, Hex 162. (TIF) [file pone.0184968.s006.tif]

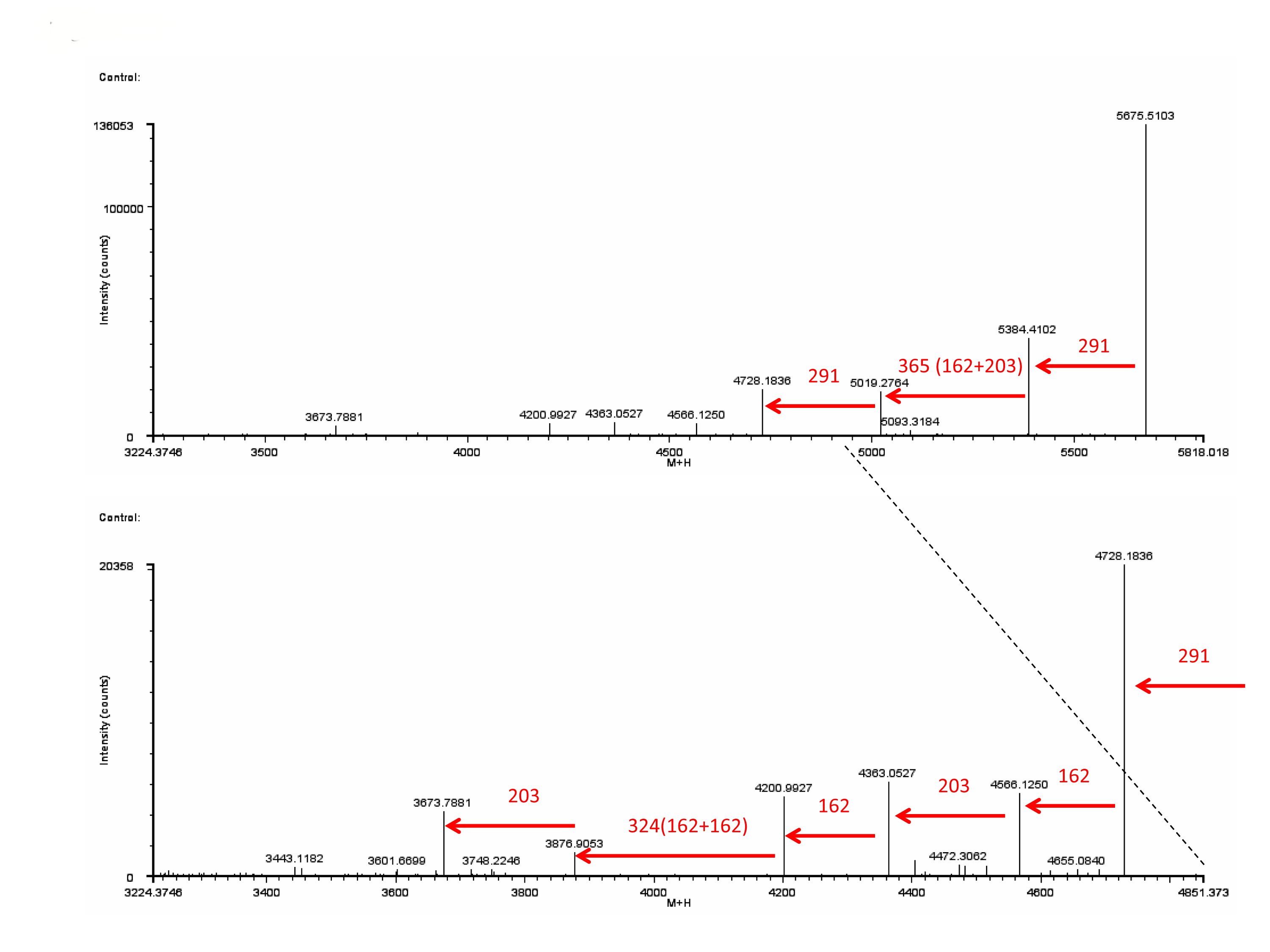

Supplement: S7 Fig — The precursor ion 5675.5103 ([M+H]+) was successively fragmented due to the loss of 2 NeuAc, 4 HexNAc and 5 Hex (glycan mass 2204.7724) Monosaccharid masses: NeuAc 291, HexNAc 203, Hex 162. (TIF) [file pone.0184968.s007.tif]

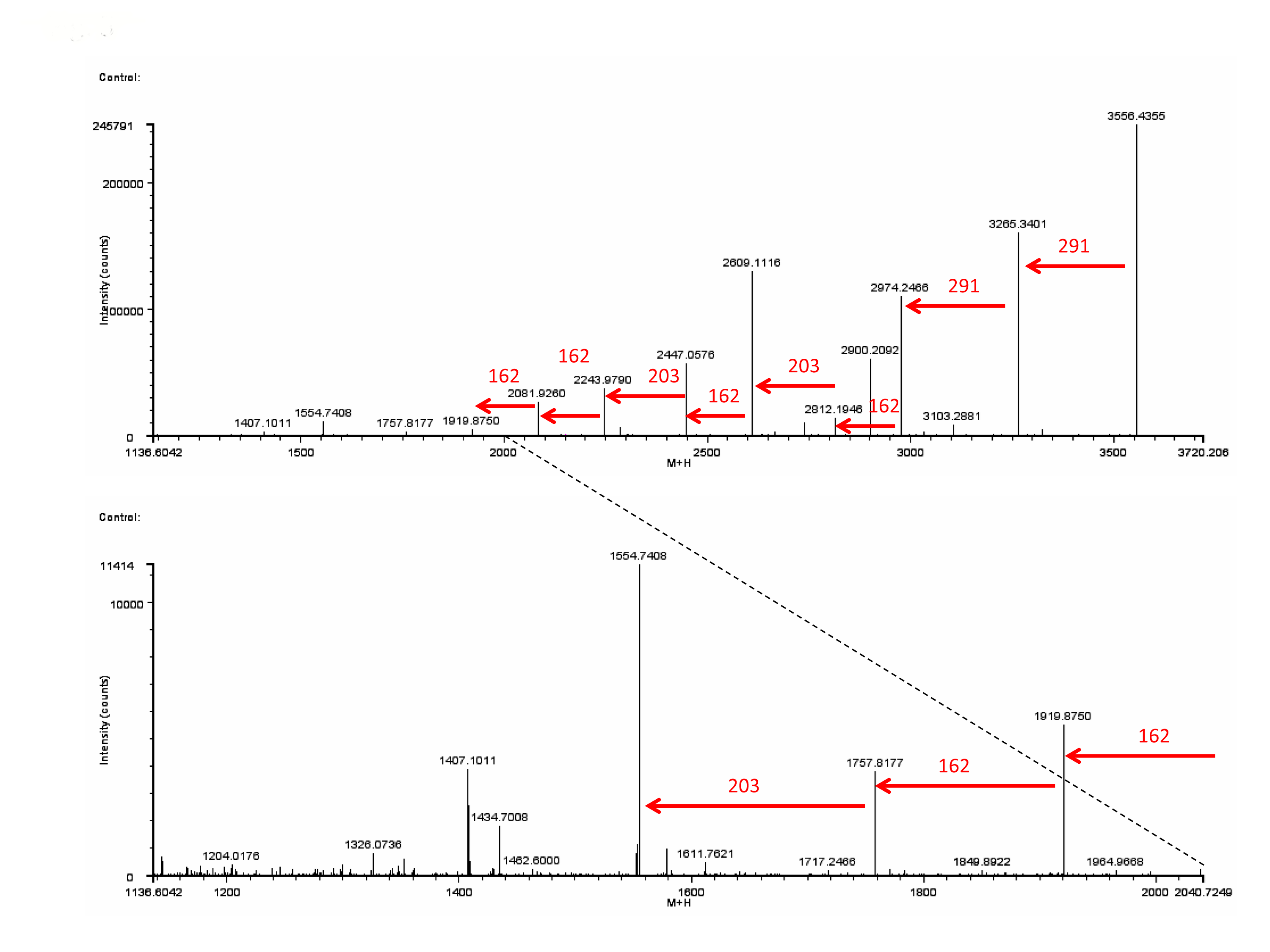

Supplement: S8 Fig — The precursor ion 3556.4355 ([M+H]+) was successively fragmented due to the loss of 2 NeuAc, 4 HexNAc and 5 Hex (glycan mass 2204.7724) Monosaccharid masses: NeuAc 291, HexNAc 203, Hex 162. (TIF) [file pone.0184968.s008.tif]

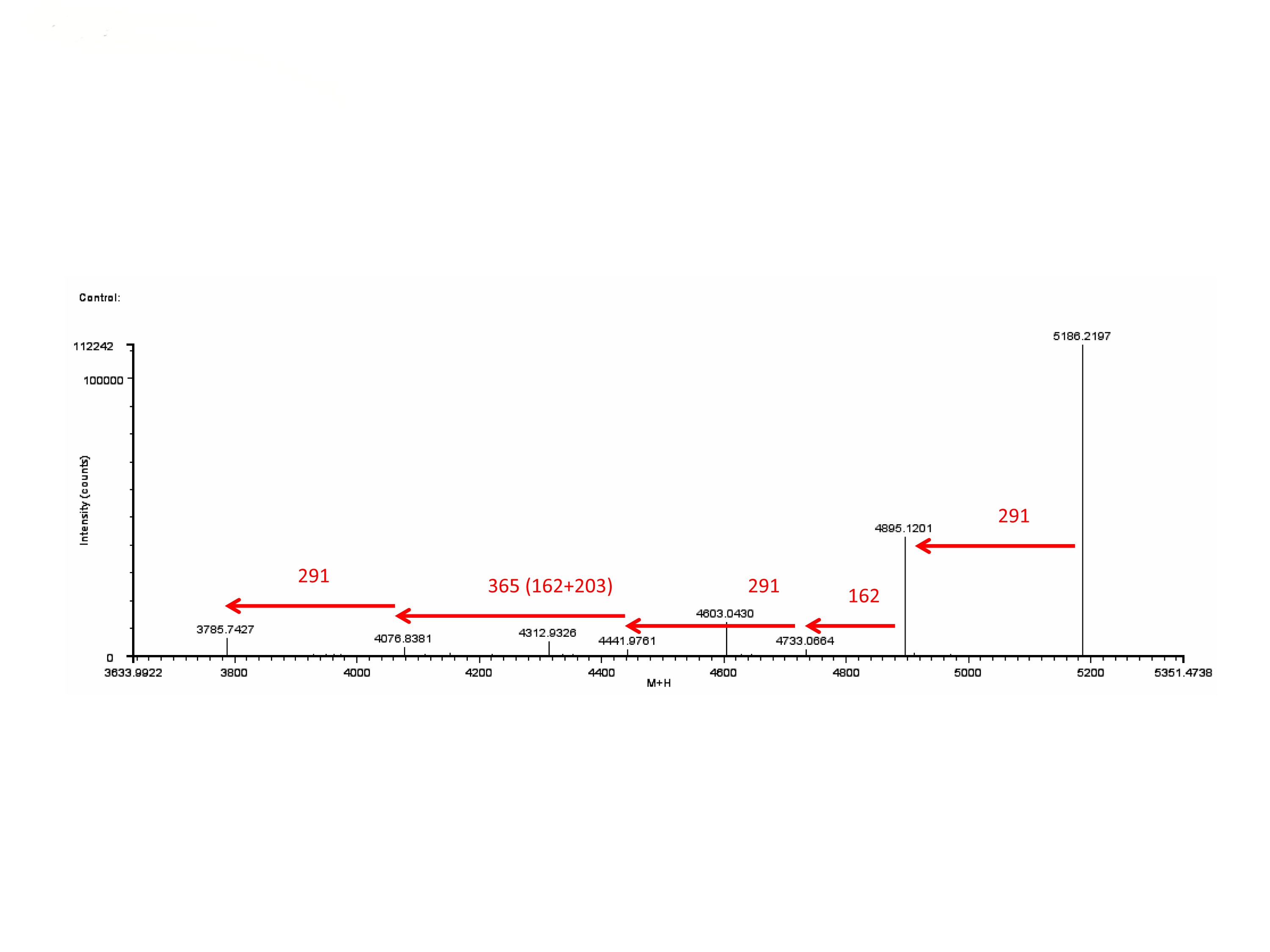

Supplement: S9 Fig — The precursor ion 5186.2197([M+H]+) was successively fragmented due to the loss of 3 NeuAc, 5 HexNAc and 6 Hex (glycan mass 2861.0000) Monosaccharid masses: NeuAc 291, HexNAc 203, Hex 162. (TIF) [file pone.0184968.s009.tif]
