# Supplementary material for: Purification of rabbit serum histidine-proline-rich glycoprotein via preparative gel electrophoresis and characterization of its glycosylation patterns
Source: PLoS One. 2017 Sep 21;12(9):e0184968. doi: 10.1371/journal.pone.0184968 (PMC5608300; doi:10.1371/journal.pone.0184968)
Supplement: S1 Table — (PDF) [file pone.0184968.s010.pdf]

ESI-MS Result of the Main His Trap HP Elution Fraction

OK	Accession	Entry	Description	mW (Da)	pI (pH)	PLGS Score	Peptides	Theoretical Peptides	Coverage (%)	Precursor RMS Mass Error (ppm)	Products	Modified Peptides	Products RMS Mass Error (ppm)	Products RMS RT Error (min)	Amount (fmol)	Amount (ngrams)
2	ALBU_RABIT	P49065	Serum albumin OS=Oryctolagus cuniculus GN=ALB PE=1 SV=2	68865	5,8022	4362,288	29	37	29,1118	1,4812	323	0	6,3188	0,00821612	128,13	9,0852
2	HRG_RABIT	Q28640	Histidine-rich glycoprotein (Fragment) OS=Oryctolagus cuniculus GN=HRG PE=1 SV=1	58840	7,2305	2481,285	26	15	14,4487	1,3243	178	0	5,7438	0,00704465	252,751	15,0686
2	A1AF_RABIT	P23035	Alpha-1-antiproteinase F OS=Oryctolagus cuniculus PE=1 SV=1	45838	5,8081	366,8674	3	16	10,8959	0,9512	32	0	6,6215	0,00784888	9,729	0,4479
