# Supplementary material for: Purification of rabbit serum histidine-proline-rich glycoprotein via preparative gel electrophoresis and characterization of its glycosylation patterns
Source: PLoS One. 2017 Sep 21;12(9):e0184968. doi: 10.1371/journal.pone.0184968 (PMC5608300; doi:10.1371/journal.pone.0184968)
Supplement: S2 Table — (PDF) [file pone.0184968.s011.pdf]

In-Gel-Digest Result of 55 kDa band (lane 2) of S1 Fig

OK	Accession	Entry	Description	mW (Da)	pI (pH)	PLGS Score	Peptides	Theoretical Peptides	Coverage (%)	Precursor RMS Mass Error (ppm)	Products	Modified Peptides	Products RMS Mass Error (ppm)	Products RMS RT Error (min)	Amount (fmol)	Amount (ngrams)
2	ALBU_RABIT	P49065	Serum albumin OS=Oryctolagus cuniculus GN=ALB PE=1 SV=2	68865	5,8022	1833,083	33	55	56,5789	1,6016	256	0	5,5645	0,00537885	49,543	3,5129
