# Supplementary material for: Purification of rabbit serum histidine-proline-rich glycoprotein via preparative gel electrophoresis and characterization of its glycosylation patterns
Source: PLoS One. 2017 Sep 21;12(9):e0184968. doi: 10.1371/journal.pone.0184968 (PMC5608300; doi:10.1371/journal.pone.0184968)
Supplement: S3 Table — (PDF) [file pone.0184968.s012.pdf]

**ESI-MS Result of the lyophilized protein after preparative gel electrophoresis**

| OK | Accession | Entry | Description | mW (Da) | pI (pH) | PLGS Score | Peptides | Theoretical Peptides | Coverage (%) | Precursor RMS Mass Error (ppm) | Products | Modified Peptides | Products RMS Mass Error (ppm) | Products RMS RT Error (min) | Amount (fmol) | Amount (ngrams) |
|---|---|---|---|---|---|---|---|---|---|---|---|---|---|---|---|---|
| 2 | HRG_RABIT | Q28640 | Histidine-rich glycoprotein (Fragment) OS=Oryctolagus cuniculus GN=HRG PE=1 SV=1 | 58840 | 7,2305 | 21140,92 | 56 | 36 | 53,2319 | 1,5146 | 765 | 0 | 5,5339 | 0,01974084 | 4499,55 | 268,2554 |
| 2 | ALBU_RABIT | P49065 | Serum albumin OS=Oryctolagus cuniculus GN=ALB PE=1 SV=2 | 68865 | 5,8022 | 1339,797 | 26 | 55 | 49,0132 | 0,8856 | 205 | 0 | 10,0398 | 0,02694459 | 51,253 | 3,6341 |
| 2 | A1AF_RABIT | P23035 | Alpha-1-antiproteinase F OS=Oryctolagus cuniculus PE=1 SV=1 | 45838 | 5,8081 | 156,9435 | 7 | 28 | 21,0654 | 8,308 | 47 | 0 | 12,7009 | 0,02678212 | 10,3 | 0,4742 |
