# Supplementary material for: Purification of rabbit serum histidine-proline-rich glycoprotein via preparative gel electrophoresis and characterization of its glycosylation patterns
Source: PLoS One. 2017 Sep 21;12(9):e0184968. doi: 10.1371/journal.pone.0184968 (PMC5608300; doi:10.1371/journal.pone.0184968)
Supplement: S4 Table — (PDF) [file pone.0184968.s013.pdf]

nanoLC-ESI-MS/MS results from the tryptic digest of HPRG after PNGase F treatment

Peptide	Start AS	End AS	Modifiers	Calculated Peptide Mass (Da)	RT (Min)	m/z	Charge State	Observed Peptide Mass (Da)	Intensity (Counts)	Mass Error (ppm)	b/y Fragment Ion List
TTKPLAEK	26	33		886,5124	12	444,2628	2	886,5097	23983	-3	b6;y2;y5;y6;y7
ALDLINK	34	40		785,4647	17,1	393,7401	2	785,4644	406621	-0,4	b2;b3;b4;b5;y1;y2;y3;y4;y5;y6
RDGYLFQLLR	43	52		1279,7037	20,9	427,5751	3	1279,7014	669338	-1,8	b1;b2;b3;b4;b5;b6;b7;b9;y1;y2;y3;y4;y5;y6;y8;y9
DGYLFQLLR	44	52		1123,6025	22,8	562,8092	2	1123,6025	222346	0	b2;b3;b4;b5;b6;y1;y2;y3;y4;y5;y6;y7
VADAHLDGAESATVYYLVLDVK	53	74		2348,1899	22	783,737	3	2348,1873	1249649	-1,1	b2;b3;b4;b5;b7;b8;b9;b10;b11;b12;b13;b14;b15;b16;b17;b18;b19;b20;b21;y1;y2;y3;y4;y5;y6;y7;y8;y9;y10;y11;y12;y13;y19;y20;y21
ETDCSVLSR	75	83	Carbamidomethyl C(1)	1065,4761	14,6	533,7455	2	1065,4751	441269	-0,9	b4*;b5*;b6*;y1;y2;y3;y4;y5;y6*;y7*;y8*
KHWEDCDPDLTK	84	95	Carbamidomethyl C(1)	1542,6772	14,4	515,233	3	1542,675	532587	-1,4	b1;b2;b3;b4;b5;b6*;b7*;b9*;b10*;b11*;y1;y2;y3;y4;y5;y6;y7*;y8*;y9*;y10*;y11*
HWEDCDPDLTK	85	95	Carbamidomethyl C(1)	1414,5824	15,3	708,298	2	1414,5802	79168	-1,6	b2;b4;b5*;b6*;b8*;b9*;b10*;y2;y3;y4;y5;y6;y8*;y9*;y10*
RPSLDVIGQCK	96	106	Carbamidomethyl C(1)	1271,6656	16	636,8397	2	1271,6635	570554	-1,7	b2;b3;b4;b5;b6;b7;b8;b9;b10*;y2*;y3*;y4*;y5*;y6*;y7*;y9*;y10*
YSDEYQTLR	112	120		1173,5303	15,3	587,7724	2	1173,5289	711336	-1,2	b1;b2;b3;b4;b5;b6;b7;b8;y1;y2;y3;y4;y5;y6;y7;y8
LNDFDCTTSSVSSALANTK	121	139	Carbamidomethyl C(1),Deglycosylation of N 125	2029,9263	18,6	1015,9696	2	2029,9233	855896	-1,5	b2*;b3;b5*;b6*;b8*;b9*;b11*;b13*;b14*;b15*;b16*;b18*;y2;y3;y4;y5;y6;y7;y8;y9;y10;y11;y12;y14*;y15*;y16*;y17*;y18*
DSPVLFDFIEDTEPFRK	140	156		2053,9995	23,2	685,6737	3	2053,9973	768780	-1,1	b2;y2;y3;y4;y6;y7;y8;y11;y12;y13;y14;y16
DSPVLFDFIEDTEPFR	140	155		1925,9047	23,4	963,9597	2	1925,9034	296587	-0,7	b5;b6;b8;b9;b10;b12;b13;y2;y3;y4;y5;y6;y7;y8;y10;y11;y12
ALEVYK	161	166		721,401	15,1	361,708	2	721,4002	277183	-1,1	b2;b3;b4;b5;y1;y2;y3;y4;y5
ALEVYKSESEAYASFR	161	176		1848,8894	17,9	617,3044	3	1848,8895	3386	0,1	b2;y3;y4;y6;y10;y14
SESEAYASFR	167	176		1145,4989	15,6	573,7563	2	1145,4967	674885	-1,9	b2;b3;b4;b5;b6;b7;y2;y3;y4;y5;y6;y7;y8;y9
TNYYVDFSVR	192	201		1262,5931	19	632,3034	2	1262,5909	819073	-1,7	b2;b3;b4;b6;b7;b8;y1;y2;y3;y4;y5;y6;y7;y8;y9
SHFHR	206	210		682,33	5	342,1733	2	682,3308	1186	1,2	b2;y1;y3
HPAFGFCR	211	218	Carbamidomethyl C(1)	990,4494	15,9	496,2319	2	990,448	238324	-1,4	b2;b3;b7*;y2*;y3*;y4*;y5*;y6*;y7*
ADLSFDVEASNLENPEDVIISCEVFNFEEHGDISGFRPHLGK	219	260	Carbamidomethyl C(1),Deglycosylation of N 250	4732,2026	23	947,4489	5	4732,2046	543359	0,4	b3;b4;b6;b7;b8;b11;b12;b14;b18;y3;y5;y10;y16*;y18*;y19*;y21*;y24*;y25*;y26*;y28*;y29*;y30*;y31*;y32*;y33*;y34*;y35*
TPLGTDGSR	261	269		902,4457	13,2	452,2303	2	902,4448	353662	-1	b2;b3;b4;b5;y1;y2;y3;y4;y5;y6;y7;y8

FGCPPPQEGEDFSEGPPSQGGT PPLSPPSGPR	280	311	Carbamidomethyl C(1)	3261,4673	18,3	1088,1628	3	3261,4646	926903	-0,8	b3*;b4*;b5*;b7*;b8*;b10*;b11*;b12*;b13*;b14*;b16*;b18*;b19*;b22*;b23*;b24*;b25*;b26*;b27*;b29*;b30*;b31*;y3;y5;y6;y7;y8;y9;y10;y11;y13;y14;y15;y16;y17;y18;y19;y20;y21;y24;y25;y27;y28;y29;y30*
HRPFGTDETHR	314	324	Deglycosylation of N 320	1351,6381	12,3	451,5537	3	1351,6372	305914	-0,7	b1;b2;b4;b5;b7*;b8*;b9*;b10*;y1;y2;y3;y4;y5*;y6*;y7*;y8*;y9*
FPHHR	325	329		692,3506	11,2	347,1825	2	692,3491	4120	-2,2	y2;y3;y4
EGPQDLHQHGHGPPPK	444	459		1729,8284	12,4	433,4644	4	1729,8257	612636	-1,6	b1;b2;b5;b9;b10;b11;b12;b13;y1;y2;y3;y4;y5;y6;y7;y8;y9;y10;y11;y12;y13;y14;y15
EGPQDLHQHGHGPPPKHPGK	444	463		2149,0564	12	538,2704	4	2149,05	9985	-3	y2;y3;y6;y7;y16;y18
GHFPFHWR	469	476		1082,5199	17,2	361,8478	3	1082,5195	186230	-0,4	b2;b3;b4;y2;y3;y4;y5;y6;y7
RIGSVYQLPPLQK	477	489		1497,8667	18,2	500,2963	3	1497,865	98886	-1,1	b4;b5;b7;b8;b11;b12;y2;y3;y5;y6;y7
IGSVYQLPPLQK	478	489		1341,7656	19	671,8892	2	1341,7625	1189257	-2,3	b1;b2;b3;b4;b6;b7;b8;b9;b10;b11;y2;y3;y4;y5;y6;y7;y8;y9;y10;y11
GEVLPLPEANFPSFSLR	490	506		1871,9781	22,9	936,9967	2	1871,9775	1208434	-0,3	b2;b3;b4;b5;b6;b8;b9;b10;b11;b14;y3;y4;y6;y7;y9;y11;y12;y13;y14;y15
GEVLPLPEANFPSFSLRDHTHPL KPEIQPFPQVASER	490	526	Deglycosylation of N 507	4179,1543	21,3	836,8382	5	4179,1514	3906	-0,7	b3;b4;y7;y31*
DHTHPLKPEIQPFPQVASER	507	526	Deglycosylation of N 507	2325,1865	17,4	582,3043	4	2325,1855	1687316	-0,4	b2*;b3*;b4*;b5*;b6*;b7*;b8*;b9*;b10*;b11*;b12*;b13*;b14*;b15*;b16*;b19*;y1;y2;y3;y4;y5;y6;y7;y8;y9;y10;y12;y13;y16;y17;y18;y19
CPEEFNGEFAQLSK	527	540	Carbamidomethyl C(1)	1654,7297	18,6	828,369	2	1654,7222	485418	-4,5	b2*;b3*;b4*;b5*;b6*;b9*;b10*;b12*;y2;y3;y4;y5;y6;y7;y8;y10;y11;y12;y13
FFPSTFPK	541	548		969,496	18,8	485,7558	2	969,4957	964136	-0,3	b2;b6;y1;y2;y3;y4;y5;y6;y7
