# Supplementary material for: Purification of rabbit serum histidine-proline-rich glycoprotein via preparative gel electrophoresis and characterization of its glycosylation patterns
Source: PLoS One. 2017 Sep 21;12(9):e0184968. doi: 10.1371/journal.pone.0184968 (PMC5608300; doi:10.1371/journal.pone.0184968)
Supplement: S5 Table — (PDF) [file pone.0184968.s014.pdf]

nanoLC-ESI-MS/MS results from the Asp-N digest of HPRG after PNGase F treatment

Peptide	Start AS	End AS	Modifiers	Calculated Peptide Mass (Da)	RT (Min)	m/z	Charge State	Observed Peptide Mass (Da)	Intensity (Counts)	Mass Error (ppm)	b/y Fragment Ion List
DCKTTKPLAEKALDLINKWRRDGYLFQLLRVA	23	54	Carbamidomethyl C(1)	3831,0981	23	767,2258	5	3831,0894	6735	-2,3	b2*;b3*;b4*;b5*;b25*;y2;y3;y4;y5;y6;y17;y22;y25;y28
DCKTTKPLAEKALDLINKWRRDGYLFQLLRVA	23	54	Carbamidomethyl C(1)	3831,0981	23	548,3064	7	3831,0894	1861	-2,3	b2*;b3*;b4*;b5*;b25*;y2;y3;y6;y17;y28
DLINKWRRDGYLFQLLRVA	36	54		2375,3225	22,4	594,8384	4	2375,3218	5464	-0,3	b9;b14;y4;y5;y6
DGAESATVYYLVL	59	71		1399,6871	23,3	700,8516	2	1399,6873	38402	0,1	b2;b3;b4;b5;b6;b7;b8;b10;b12;y1;y2;y3;y4
DVKETDCSVLSRKHWE	72	87	Carbamidomethyl C(1)	1987,9421	15,4	497,993	4	1987,9401	384424	-1	b2;b3;b4;b5;b6;b7*;y2;y3;y4;y6;y7;y8;y9;y10*;y12*;y13*;y14*;y15*
DCSVLSRKHWE	77	87	Carbamidomethyl C(1)	1415,6616	15,3	472,8943	3	1415,6591	117017	-1,8	b2*;b3*;y1
DVIGQCKVIATRYS	100	113	Carbamidomethyl C(1)	1608,8293	16,6	537,2825	3	1608,8237	674497	-3,5	b2;b3;b4;b5;b6*;b7*;b9*;b10*;b13*;y2;y3;y4;y5;y6;y7;y8;y9*;y10*;y11*;y12*;y13*
DVIGQCKVIATRYSDEYQTLRLN	100	122	Carbamidomethyl C(1)	2741,3806	18,9	686,3531	4	2741,3809	6922	0,1	b7*;b15*;y4;y5
DEYQTLRLN	114	122		1150,5619	17,3	576,2875	2	1150,5592	463251	-2,3	b1;b2;b3;b4;b5;b6;b7;b8;y1;y2;y3;y4;y5;y6;y7
DFDCTTSSVSSALANTK	123	139	Carbamidomethyl C(1),Deglycosylation of N 125	1802,7993	18	902,408	2	1802,8	11187	0,4	b14*;y2;y3;y5;y8;y10;y12;y13
DCTTSSVSSALANTK	125	139	Carbamidomethyl C(1),Deglycosylation of N 125	1540,704	16,2	771,3582	2	1540,7006	584916	-2,2	b2*;b3*;b4*;b5*;b6*;b7*;b8*;b9*;b10*;b11*;b12*;b13*;b14*;y1;y2;y3;y4;y5;y6;y7;y8;y9;y10;y11;y12;y13;y14*
DSPVLF	140	145		676,3432	21,2	677,3496	1	676,3417	278693	-2,2	b2;b3;b4;b5;y1;y2;y3;y4;y5
DFIEDTEPFRKSA	146	158		1553,7362	18,3	777,8752	2	1553,7345	712815	-1,1	b2;b3;b4;b5;b6;b7;b10;b11;b12;y3;y4;y5;y6;y7;y8;y9;y10;y11;y12
DFIE	146	149		522,2325	17,4	523,2392	1	522,2313	71535	-2,3	b2;b3;y1;y2
DTEPFRKSA	150	158		1049,5142	13,4	525,7656	2	1049,5153	145467	1	b2;b3;b7;b8;y2;y3;y4;y5;y6;y7;y8
DKALEVYKSESEAYASFRV	159	177		2191,0796	19	731,3673	3	2191,0781	426987	-0,7	b2;b3;b4;b5;b6;b7;b10;b11;b12;b13;b14;b15;b18;y2;y3;y4;y5;y6;y7;y9;y10;y11;y12;y13;y16;y17
DRVERVTRVKGGERTNYYV	178	196		2296,2036	14,2	575,0588	4	2296,2034	187196	-0,1	b2;b4;b5;b8;b10;b14;b16;b17;b18;y1;y2;y9;y15
DRVERVTRVKGGERTNYYVDFSVR	178	201		2900,5005	16	581,1074	5	2900,4976	43984	-1	b4;b16;b20;y2;y3;y4;y23
DFSVR	197	201		622,3075	14,9	312,1615	2	622,3071	22353	-0,6	y1;y2;y3;y4
DCSRSHFHRHPAFGFCRA	202	219	Carbamidomethyl C(2), Deglycosylation of N 202	2243,9966	14,3	562,007	4	2243,9961	105835	-0,2	b2*;b5*;b6*;b7*;b8*;b9*;b10*;b12*;b14*;y2;y3*;y4*;y5*;y6*;y7*;y8*;y11*;y12*
DLSF	220	223		480,222	19,4	481,2295	1	480,2216	111379	-0,8	b2;b3;y1;y2;y3
DLSFDVEASNLENPE	220	234		1677,7369	21,3	839,8764	2	1677,7369	12269	0	b2;b3;b4;y5;y11
DVEASNLENPE	224	234		1215,5255	15,9	608,77	2	1215,524	159415	-1,2	b2;b3;b4;b5;b6;b7;b8;b9;y1;y2;y3;y4;y6;y8;y9
DVIISCEVFNFEEHG	235	249	Carbamidomethyl C(1)	1793,7931	21,8	897,9041	2	1793,7922	280659	-0,5	b2;b3;b4;b5;b6*;b9*;b10*;b11*;b12*;b13*;b14*;y2;y3;y4;y5;y6;y7;y8;y9;y10*;y11*;y12*;y13*
DISGFRPHLGKTPLGTDGSR	250	269	Deglycosylation of N 250	2110,0918	17,1	528,5302	4	2110,0889	899898	-1,4	b2;b3;b4;b6;b8;b9;b11;b12;b13;b14;b17;y1;y2;y3;y4;y5;y6;y8;y9;y10;y11;y12;y14;y17;y18;y19
DISGFRPHLGKTPLGT	250	265	Deglycosylation of N 250	1694,9104	18,2	565,9778	3	1694,9098	97799	-0,4	b2;b3;b6;b8;b10;y2;y4;y5;y7;y8;y10;y12;y13;y14
DHHHPHKPHKFGCPPPQEGE	270	289	Carbamidomethyl C(1)	2367,0715	12,1	592,7751	4	2367,0686	162475	-1,2	b4;b7;b13*;b14*;b15*;b17*;b18*;b19*;y1;y2;y3;y5;y6;y7;y13*;y16*;y17*;y18*;y19*

DHHHPHKPHKFGCPPPQEGEDFSEGPPSQGGTPPLSPPSGPRCRHRPFGT	270	319	Carbamidomethyl C(2)	5523,5664	15,5	691,4537	8	5523,5664	55660	0	b2;b4;b14*;b15*;b17*;b19*;b20*;b21*;b22*;b25*;b30*;y15*;y17*;y18*;y20*;y21*;y22*;y24*;y25*;y26*;y30*
DFSEGPPSQGGTPPLSPPSGPRCRHRPFGTDETHRFPHHR	290	329	Carbamidomethyl C(1),Deglycosylation of N 320	4487,1226	15,2	748,8615	6	4487,1211	178516	-0,3	b2;b3;b4;b5;b6;b11;b12;b21;y4;y5;y8;y9;y14;y16;y24*;y25*;y26*;y27*;y28*;y29*;y31*;y34*;y35*;y36*;y38*
DFSEGPPSQGGTPPLSPPSGPRCRHRPFGT	290	319	Carbamidomethyl C(1)	3174,5054	16,7	794,6335	4	3174,5022	96014	-1	b2;b3;b5;b9;b12;y6;y14*;y15*;y17*;y18*;y21*;y22*;y23*;y24*;y25*;y26*;y27*;y28*
DETHRFPHHR	320	329	Deglycosylation of N 320	1330,6279	11,5	444,5496	3	1330,625	55742	-2,2	b2;b5;b6;b8;y1;y2;y3;y4;y5;y6;y7;y8;y9
DHGPCDPPSHKEGPQ	433	447	Carbamidomethyl C(1)	1656,6951	11,8	553,2386	3	1656,6919	331633	-1,9	b2;b3;b4;b5*;b6*;b7*;b8*;b9*;b13*;y2;y3;y4;y5;y6;y7;y8;y9;y11*;y12*;y13*;y14*
DPPSHKEGPQ	438	447		1090,5043	10,4	546,2593	2	1090,5027	7258	-1,5	y7;y8;y9
