# Supplementary material for: Purification of rabbit serum histidine-proline-rich glycoprotein via preparative gel electrophoresis and characterization of its glycosylation patterns
Source: PLoS One. 2017 Sep 21;12(9):e0184968. doi: 10.1371/journal.pone.0184968 (PMC5608300; doi:10.1371/journal.pone.0184968)

Preparative Gel Electrophoresis

Custom-made gel chambers with the following characteristics:

- Glass plates: 20 x 20 cm, thickness: 4 mm
- Notched glass plates: notch of 16 x 2 cm, thickness: 4 mm
- Spacer: 20 x 1 x 0.15 cm (1.5 mm Spacer)
- Separation Gel: 18 x 15 cm x 0.15 cm
- Stacking Gel: 18 x 2 cm x 0.15 cm
- Buffer tanks: cathodic tank: ca. 500 ml anodic tank: ca. 250 ml
- Sample Slot: either single slot generated with an inverted mounted comb;
  however it is also possible (and was done in this work) to overlay the poured stacking gel with a layer of isopropanol for polymerization (like it is done after pouring the separation gel); take care to remove the isopropanol completely
  sample is then loaded onto the stacking gel

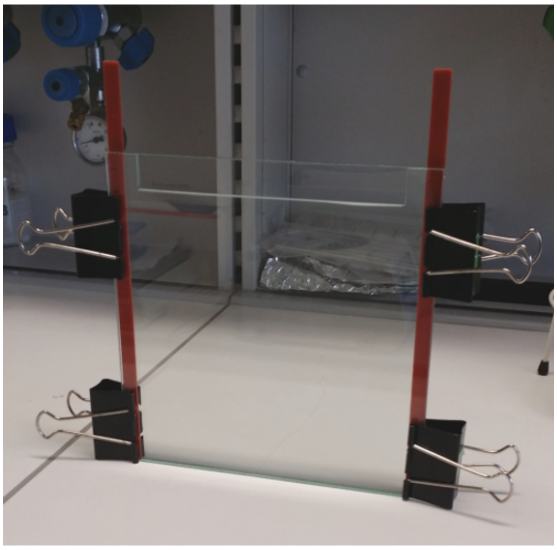

	**Separation Gel (10%)**			**Stacking Gel (5%)**	
	1 mm Spacer	**1.5 mm Spacer**		1 mm Spacer	**1.5 mm Spacer**
**H_2O_{dd}**	13 ml	**19.5 ml**	**H_2O_{dd}**	6.95 ml	**10.425 ml**
**2M Tris, pH 8.8**	8 ml	**12 ml**	**1M Tris, pH 6.8**	1.25 ml	**1.875 ml**
**30% Acrylamid**	10.7 ml	**16.0 ml**	**30% Acrylamid**	1.67 ml	**2.500 ml**
**10% SDS**	320 µl	**480 µl**	**10% SDS**	100 µl	**150 µl**
**10% APS**	150 µl	**225 µl**	**10% APS**	50 µl	**75 µl**
**TEMED**	15 µl	**22.5 µl**	**TEMED**	5 µl	**7.5 µl**

Calculation for different thicknesses, e.g. a 2 mm spacer: use the respective volume of the 1 mm spacer recipe and multiply by 2

Running Conditions:

- Voltage: 60 – 70 constant V; current: 30 mA start, ca. 8 mA end (usually start in the afternoon until next morning; if needed: 200 V in the next morning if dye front has not completely migrated to the anodic side) (approximately about 1500 $V \cdot h$ are needed for the dye front to migrate to the anodic site, thus the needed voltage for a certain time can also be calculated)
- Temperature: room temperature (without a cooling device)
- Electrophoresis type: according to Laemmli, but no thermic denaturation of sample and no adding of reducing agent
- Running buffer: 25 mM Tris, 192 mM Glycine, 0.1% SDS
- Staining: Zinc-imidazole staining (as stated in Material and Method section (procedure described in Step 2 in Ref. 29))
- band corresponding to protein of interest is cut out and is sliced into small pieces

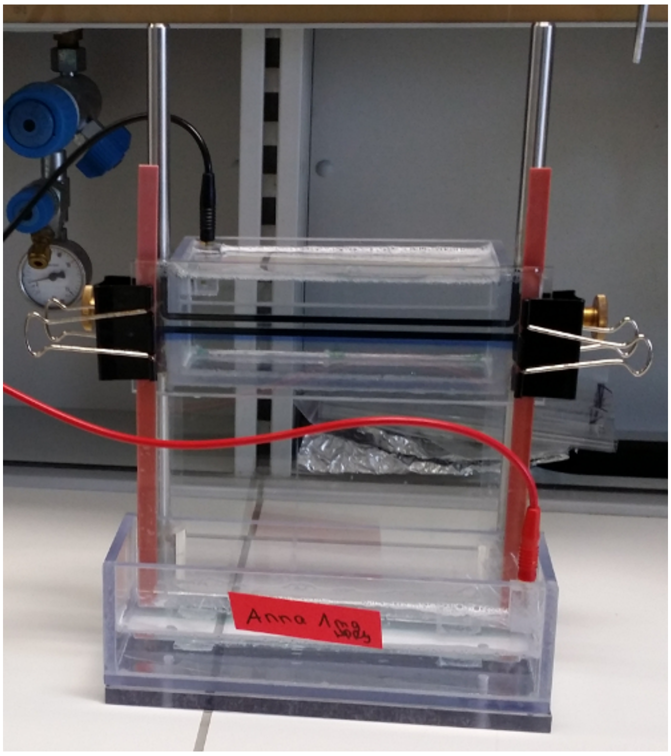

Electroelution (EE)

- Electro elution chambers: Custom-made chambers that correspond to the “bridge type electro-eluter” type (see: Holger Seelert and Frank Klause: Preparative isolation of protein complexes and other bioparticles by elution from polyacrylamide gels, Electrophoresis, 2008).
- Dialysis membrane: Serva 3.5 kDa cutoff

- Gel slices are filled in the broad part of the electroelution chamber (cathodic side)
- Elution buffer: Tris-Glycine PAGE running buffer: 25 mM Tris, 192 mM Glycine, 0.1% SDS + additionally 2% SDS added on the cathodic side (preparation of 40 ml running buffer + 10 ml of 10% SDS; pipette on the cathodic side of the electroelution chamber and take care that the bridge is also filled with buffer, otherwise there will be no current flow
- Voltage: 70V corresponding to ca. 2-3 mA per chamber (start) dropping to ca. 1 mA/chamber at the end
- Temperature: room temperature
- Time: ca. 15h

- after finishing EE the protein solution is taken out of the anodic side of the EE chamber via a HPLC syringe (use a plan tip / do not use a sharp needle to not damaging the membrane)
- membrane should also be washed a few times with EE buffer to remove residual protein)

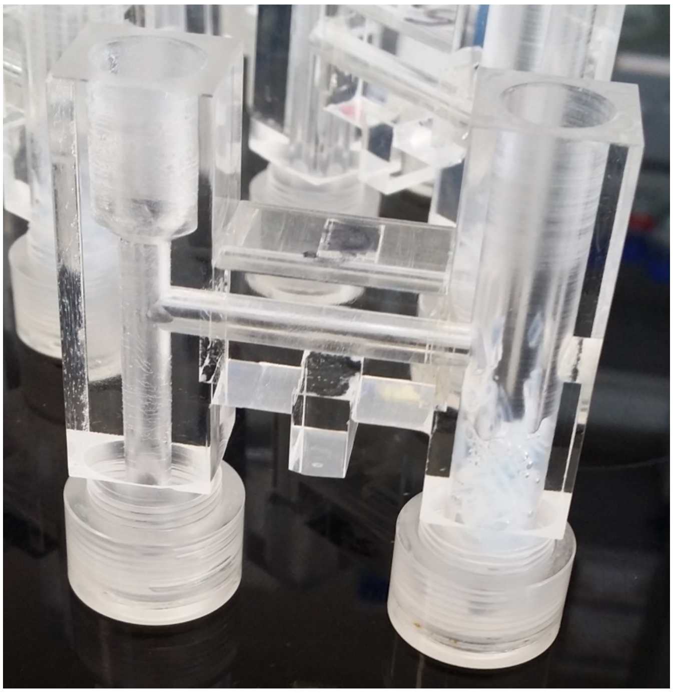

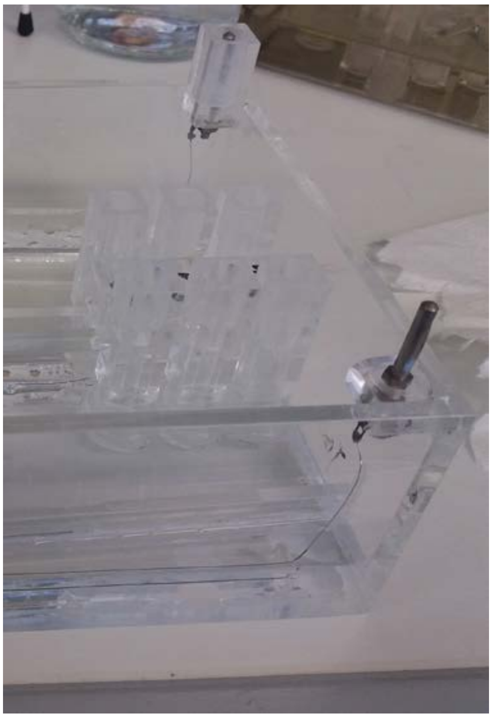

Supplement: S1 Appendix — (PDF) [file pone.0184968.s015.pdf]
